# Efficient conservative ADER schemes based on WENO reconstruction and space-time predictor in primitive variables

**DOI:** 10.1186/s40668-015-0014-x

**Published:** 2016-01-13

**Authors:** Olindo Zanotti, Michael Dumbser

**Affiliations:** Laboratory of Applied Mathematics, Department of Civil, Environmental and Mechanical Engineering, University of Trento, Via Mesiano 77, Trento, 38123 Italy

**Keywords:** high order WENO reconstruction in primitive variables, ADER-WENO finite volume schemes, ADER discontinuous Galerkin schemes, AMR, hyperbolic conservation laws, relativistic hydrodynamics and magnetohydrodynamics, Baer-Nunziato model

## Abstract

We present a new version of conservative ADER-WENO finite volume schemes, in which both the high order spatial reconstruction as well as the time evolution of the reconstruction polynomials in the local space-time predictor stage are performed in *primitive* variables, rather than in conserved ones. To obtain a conservative method, the underlying finite volume scheme is still written in terms of the cell averages of the conserved quantities. Therefore, our new approach performs the spatial WENO reconstruction *twice*: the *first* WENO reconstruction is carried out on the known *cell averages* of the conservative variables. The WENO polynomials are then used at the cell centers to compute *point values* of the *conserved variables*, which are subsequently converted into *point values* of the *primitive variables*. This is the only place where the conversion from conservative to primitive variables is needed in the new scheme. Then, a *second* WENO reconstruction is performed on the point values of the primitive variables to obtain piecewise high order reconstruction polynomials of the primitive variables. The reconstruction polynomials are subsequently evolved in time with a *novel* space-time finite element predictor that is directly applied to the governing PDE written in *primitive form*. The resulting space-time polynomials of the primitive variables can then be directly used as input for the numerical fluxes at the cell boundaries in the underlying *conservative* finite volume scheme. Hence, the number of necessary conversions from the conserved to the primitive variables is reduced to just *one single conversion* at each cell center. We have verified the validity of the new approach over a wide range of hyperbolic systems, including the classical Euler equations of gas dynamics, the special relativistic hydrodynamics (RHD) and ideal magnetohydrodynamics (RMHD) equations, as well as the Baer-Nunziato model for compressible two-phase flows. In all cases we have noticed that the new ADER schemes provide *less oscillatory solutions* when compared to ADER finite volume schemes based on the reconstruction in conserved variables, especially for the RMHD and the Baer-Nunziato equations. For the RHD and RMHD equations, the overall accuracy is improved and the CPU time is reduced by about 25 %. Because of its increased accuracy and due to the reduced computational cost, we recommend to use this version of ADER as the standard one in the relativistic framework. At the end of the paper, the new approach has also been extended to ADER-DG schemes on space-time adaptive grids (AMR).

## Introduction

Since their introduction by Toro and Titarev (Toro et al. 2001; Titarev and Toro 2002; Toro and Titarev 2002; Titarev and Toro 2005; Toro and Titarev 2006), ADER (arbitrary high order derivatives) schemes for hyperbolic partial differential equations (PDE) have been improved and developed along different directions. A key feature of these methods is their ability to achieve uniformly high order of accuracy in space and time in a single step, without the need of intermediate Runge-Kutta stages (Pareschi et al. 2005; Pidatella et al. 2015), by exploiting the approximate solution of a Generalized Riemann Problem (GRP) at cell boundaries. ADER schemes have been first conceived within the finite volume (FV) framework, but they were soon extended also to the discontinuous Galerkin (DG) finite element framework (Dumbser and Munz 2006; Taube et al. 2007) and to a unified formulation of FV and DG schemes, namely the so-called $\mathbb{P}_{N}\mathbb{P}_{M}$ approach (Dumbser et al. 2008a). In the original ADER approach by Toro and Titarev, the approximate solution of the GRP is obtained through the solution of a conventional Riemann problem between the boundary-extrapolated values, and a sequence of linearized Riemann problems for the spatial derivatives. The required time derivatives in the GRP are obtained via the so-called Cauchy-Kowalevski procedure, which consists in replacing the time derivatives of the Taylor expansion at each interface with spatial derivatives of appropriate order, by resorting to the strong differential form of the PDE. Such an approach, though formally elegant, becomes prohibitive or even impossible as the complexity of the equations increases, especially for multidimensional problems and for relativistic hydrodynamics and magneto-hydrodynamics. On the contrary, in the modern reformulation of ADER (Dumbser et al. 2008b; Dumbser et al. 2008a; Balsara et al. 2013), the approximate solution of the GRP is achieved by first evolving the data locally inside each cell through a *local space-time discontinuous Galerkin predictor* (LSDG) step that is based on a weak form of the PDE, and, second, by solving a sequence of classical Riemann problems along the time axis at each element interface. This approach has the additional benefit that it can successfully cope with stiff source terms in the equations, a fact which is often encountered in physical applications. For these reasons, ADER schemes have been applied to real physical problems mostly in their modern version. Notable examples of applications include the study of Navier-Stokes equations, with or without chemical reactions (Hidalgo and Dumbser 2011; Dumbser 2010), geophysical flows (Dumbser et al. 2009), complex three-dimensional free surface flows (Dumbser 2013), relativistic magnetic reconnection (Dumbser and Zanotti 2009; Zanotti and Dumbser 2011), and the study of the Richtmyer-Meshkov instability in the relativistic regime (Zanotti and Dumbser 2015). In the last few years, ADER schemes have been enriched with several additional properties, reaching a high level of flexibility. First of all, ADER schemes have been soon extended to deal with non-conservative systems of hyperbolic PDE (Toro and Hidalgo 2009; Dumbser et al. 2009; Dumbser et al. 2014), by resorting to path-conservative methods (Parés and Castro 2004; Pares 2006). ADER schemes have also been extended to the Lagrangian framework, in which they are currently applied to the solution of multidimensional problems on unstructured meshes for various systems of equations, (Boscheri and Dumbser 2013; Dumbser and Boscheri 2013; Boscheri et al. 2014a; Boscheri et al. 2014b; Boscheri and Dumbser 2014). On another side, ADER schemes have been combined with Adaptive Mesh Refinement (AMR) techniques (Dumbser et al. 2013; Zanotti and Dumbser 2015), exploiting the local properties of the discontinuous Galerkin predictor step, which is applied cell-by-cell irrespective of the level of refinement of the neighbour cells. Moreover, ADER schemes have also been used in combination with discontinuous Galerkin methods, even in the presence of shock waves and other discontinuities within the flow, thanks to a novel *a posteriori* sub-cell finite volume limiter technique based on the MOOD approach (Clain et al. 2011; Diot et al. 2012), that is designed to stabilize the discrete solution wherever the DG approach fails and produces spurious oscillations or negative densities and pressures (Dumbser et al. 2014; Zanotti et al. 2015a; Zanotti et al. 2015b).

The various implementations of ADER schemes mentioned so far differ under several aspects, but they all share the following common features: they apply the local space-time discontinuous Galerkin predictor to the conserved variables, which in turn implies that, if a WENO finite volume scheme is used, the spatial WENO reconstruction is also performed in terms of the conserved variables. Although this may be regarded as a reasonable choice, it has two fundamental drawbacks. The first one has to do with the fact that, as shown by Munz (1986), the reconstruction in conserved variables provides the worst shock capturing fidelity when compared to the reconstruction performed either in primitive or in characteristic variables. The second drawback is instead related to computational performance. Since the computation of the numerical fluxes requires the calculation of integrals via Gaussian quadrature, the physical fluxes must necessarily be computed at each space-time Gauss-Legendre quadrature point. However, there are systems of equations (e.g. the relativistic hydrodynamics or magnetohydrodynamics equations) for which the physical fluxes can only be written in terms of the primitive variables. As a result, a conversion from the conserved to the primitive variables is necessary for the calculations of the fluxes, and this operation, which is never analytic for such systems of equations, is rather expensive. For these reasons it would be very desirable to have an ADER scheme in which both the reconstruction and the subsequent local space-time discontinuous Galerkin predictor are performed in primitive variables. It is the aim of the present paper to explore this possibility. It is also worth stressing that in the context of high order finite difference Godunov methods, based on traditional Runge-Kutta discretization in time, the reconstruction in primitive variables has been proved to be very successful by Del Zanna et al. (2007) in their ECHO general relativistic code (see also Bucciantini and Del Zanna 2011; Zanotti et al. 2011). In spite of the obvious differences among the numerical schemes adopted, the approach that we propose here and the ECHO-approach share the common feature of requiring a single (per cell) conversion from the conserved to the primitive variables.

The plan of the paper is the following: in Section 2 we describe the numerical method, with particular emphasis on Section 2.3 and on Section 2.4, where the spatial reconstruction strategy and the local space-time discontinuous Galerkin predictor in primitive variable are described. The results of our new approach are presented in Section 3 for a set of four different systems of equations. In Section 4 we show that the new strategy can also be extended to pure discontinuous Galerkin schemes, even in the presence of space-time adaptive meshes (AMR). Finally, Section 5 is devoted to the conclusions of the work.

## Numerical method

We present our new approach for purely regular Cartesian meshes, although there is no conceptual reason preventing the extension to general curvilinear or unstructured meshes, which may be considered in future studies.

### Formulation of the equations

We consider hyperbolic systems of balance laws that contain both conservative and non-conservative terms, i.e.

1 $$ \frac{\partial\mathbf{Q}}{\partial t}+\nabla\cdot\mathbf{F}(\mathbf{Q})+\mathbf{B}( \mathbf{Q} )\cdot \nabla\mathbf{Q}=\mathrm{S}(\mathbf{Q}) , $$

where $\mathbf{Q}\in\Omega_{\mathbf{Q}}\subset\mathbb{R}^{\nu}$ is the state vector of the *ν* *conserved variables*, which, for the typical gas dynamics equations, are related to the conservation of mass, momentum and energy. $\mathbf{F}(\mathbf{Q})=[\mathbf{ f}^{x}(\mathbf{Q}),\mathbf{ f}^{y}(\mathbf{Q}),\mathbf{ f}^{z}(\mathbf{Q})]$ is the flux tensor^1^ for the conservative part of the PDE system, while $\mathbf{B}(\mathbf{Q})=[\mathbf{B}_{x}(\mathbf{Q}),\mathbf{B}_{y}(\mathbf{Q}),\mathbf{B}_{z}(\mathbf{Q} )]$ represents the non-conservative part of it. Finally, $\mathbf{S}(\mathbf{Q})$ is the vector of the source terms, which may or may not be present. In the follow up of our discussion it is convenient to recast the system (1) in quasilinear form as

2 $$ \frac{\partial\mathbf{Q}}{\partial t}+ \mathbf{ A}(\mathbf{Q}) \cdot\nabla\mathbf{Q}= \mathbf{S}(\mathbf{Q}) , $$

where $\mathbf{A}(\mathbf{Q})=[\mathbf{A}_{x},\mathbf{A}_{y},\mathbf{A}_{z}]=\partial\mathbf{F}(\mathbf{Q} )/\partial\mathbf{Q}+\mathbf{B}(\mathbf{Q})$ accounts for both the conservative and the non-conservative contributions. As we shall see below, a proper discretization of Eq. (2) can provide the time evolution of the conserved variables **Q**, but when the *primitive variables* **V** are adopted instead, Eq. (2) translates into

3 $$\begin{aligned}& \frac{\partial\mathbf{V}}{\partial t}+ \mathbf{ C}(\mathbf{Q}) \cdot\nabla\mathbf{V}= \biggl( \frac{\partial\mathbf{Q}}{\partial\mathbf{V}} \biggr)^{-1}\mathbf{ S}(\mathbf{Q}) , \\& \quad\mbox{with } \mathbf{ C}(\mathbf{Q})= \biggl( \frac{\partial\mathbf{Q}}{\partial\mathbf{V}} \biggr)^{-1} \mathbf{ A}(\mathbf{Q} ) \biggl( \frac{\partial\mathbf{Q}}{\partial\mathbf{V}} \biggr). \end{aligned}$$

In the following we suppose that the conserved variables **Q** can always be written *analytically* in terms of the primitive variables **V**, i.e. the functions

4 $$ \mathbf{Q}=\mathbf{Q}(\mathbf{V}) $$

are supposed to be analytic for all PDE systems under consideration. On the contrary, the conversion from the conserved to the primitive variables, henceforth the *cons-to-prim conversion*, is not always available in closed form, i.e. the functions

5 $$ \mathbf{V}=\mathbf{V}(\mathbf{Q}) $$

may *not* be analytic (e.g. for relativistic hydrodynamics and magnetohydrodynamics to be discussed in Section 3.2), thus requiring an approximate numerical solution. As a result, the matrix $( \frac{\partial\mathbf{Q}}{\partial\mathbf{V} } )^{-1}$, which in principle could be simply computed as

6 $$ \biggl( \frac{\partial\mathbf{Q}}{\partial\mathbf{V}} \biggr)^{-1}= \biggl( \frac {\partial\mathbf{V}}{\partial\mathbf{Q}} \biggr) , $$

in practice it cannot be obtained in this manner, but it must be computed as

7 $$ \biggl( \frac{\partial\mathbf{Q}}{\partial\mathbf{V}} \biggr)^{-1}= \mathbf{M}^{-1} , $$

where we have introduced the notation

8 $$ \mathbf{M}= \biggl( \frac{\partial\mathbf{Q}}{\partial\mathbf{V}} \biggr) , $$

which will be used repeatedly below. Since $\mathbf{Q}(\mathbf{V})$ is supposed to be analytic, the matrix **M** can be easily computed. Equation (1) will serve us as the master equation to evolve the cell averages of the conserved variables **Q** via a standard finite volume scheme. However, both the spatial WENO reconstruction and the subsequent LSDG predictor will act on the primitive variables **V**, hence relying on the alternative formulation given by Eq. (3). The necessary steps to obtain such a scheme are described in the Sections 2.2-2.4 below.

### The finite volume scheme

In Cartesian coordinates, we discretize the computational domain Ω through space-time control volumes ${\mathcal {I}}_{ijk}=I_{ijk}\times[t^{n},t^{n}+\Delta t]=[x_{i-\frac {1}{2}},x_{i+\frac {1}{2}}]\times [y_{j-\frac {1}{2}},y_{j+\frac {1}{2}}]\times[z_{k-\frac {1}{2}},z_{k+\frac {1}{2}}]\times[t^{n},t^{n}+\Delta t]$, with $\Delta x_{i}=x_{i+\frac {1}{2}}-x_{i-\frac {1}{2}}$, $\Delta y_{j}=y_{j+\frac {1}{2}}-y_{j-\frac {1}{2}}$, $\Delta z_{k}=z_{k+\frac {1}{2}}-z_{k-\frac {1}{2}}$ and $\Delta t=t^{n+1} -t^{n}$. Integration of Eq. (1) over ${\mathcal {I}}_{ijk}$ yields the usual finite volume discretization

9 $$\begin{aligned} \bar{\mathbf{Q}}_{ijk}^{n+1} =& \bar{\mathbf{Q}}_{ijk}^{n}- \frac{\Delta t}{\Delta x_{i}} \biggl[ \bigl( \mathbf{f}^{x}_{i+\frac{1}{2},j,k} -\mathbf{f}^{x}_{i-\frac{1}{2},j,k} \bigr) \\ &{}+\frac{1}{2} \bigl({{D}}^{x}_{i+\frac{1}{2},j,k} +{{D}}^{x}_{i-\frac{1}{2},j,k} \bigr) \biggr] \\ &{} -\frac{\Delta t}{\Delta y_{j}} \biggl[ \bigl(\mathbf{ f}^{y}_{i,j +\frac{1}{2},k}- \mathbf{f}^{y}_{i,j-\frac{1}{2},k} \bigr) \\ &{}+\frac{1}{2} \bigl({{D}}^{y}_{i,j+\frac{1}{2},k}+{{D}}^{y}_{i,j-\frac{1}{2},k} \bigr) \biggr] \\ &{} -\frac{\Delta t}{\Delta z_{k}} \biggl[ \bigl(\mathbf{ f}^{z}_{i,j,k+\frac{1}{2}}- \mathbf{f}^{z}_{i,j,k-\frac{1}{2}} \bigr) \\ &{}+\frac{1}{2} \bigl({{D}}^{z}_{i,j,k+\frac{1}{2}}+{{D}}^{z}_{i,j,k-\frac{1}{2}} \bigr) \biggr] \\ &{}+ \Delta t(\bar{\mathbf{ S}}_{ijk}- \bar{ \mathbf{ P}}_{ijk}) , \end{aligned}$$

where the cell average

10 $$\begin{aligned} \bar{\mathbf{Q}}_{ijk}^{n} =&\frac{1}{\Delta x_{i}} \frac{1}{\Delta y_{j}}\frac {1}{\Delta z_{k}} \\ &{}\times\int_{x_{i-\frac{1}{2}}}^{x_{i+\frac{1}{2}}} \int_{y_{j-\frac{1}{2} }}^{y_{j+\frac{1}{2}}} \int_{z_{k-\frac{1}{2}}}^{z_{k+\frac{1}{2}}}{\mathbf{Q}}\bigl(x,y,z,t^{n} \bigr)\,dz\, dy\, dx \end{aligned}$$

is the spatial average of the vector of conserved quantities at time $t^{n}$. In Eq. (9) we recognize two different sets of terms, namely those due to the conservative part of the system (1), and those coming from the non-conservative part of it. In the former set we include the three time-averaged fluxes

11 $$\begin{aligned}& \mathbf{f}^{x}_{i+\frac{1}{2},jk} = \frac{1}{\Delta t} \frac{1}{\Delta y_{j}}\frac {1}{\Delta z_{k}} \\ & \hphantom{\mathbf{f}^{x}_{i+\frac{1}{2},jk} =}{}\times\int _{t^{n}}^{t^{n+1}} \int _{y_{j-\frac{1}{2}}}^{y_{j+\frac{1}{2}}} \int _{z_{k-\frac{1}{2} }}^{z_{k+\frac{1}{2}}} \tilde{\mathbf{ f}}^{x} \bigl({\mathbf{v}}_{h}^{-}(x_{i+\frac{1}{2} },y,z,t), \\ & \hphantom{\mathbf{f}^{x}_{i+\frac{1}{2},jk} =}{\mathbf{v} }_{h}^{+}(x_{i+\frac{1}{2}},y,z,t) \bigr) \,dz\, dy\, dt, \end{aligned}$$

12 $$\begin{aligned}& \mathbf{f}^{y}_{i,j+\frac{1}{2},k}=\frac{1}{\Delta t} \frac{1}{\Delta x_{i}}\frac {1}{\Delta z_{k}} \\ & \hphantom{\mathbf{f}^{y}_{i,j+\frac{1}{2},k}=}{}\times \int _{t^{n}}^{t^{n+1}} \int _{x_{i-\frac{1}{2}}}^{x_{i+\frac{1}{2}}} \int _{z_{k-\frac{1}{2} }}^{z_{k+\frac{1}{2}}} \tilde{\mathbf{ f}}^{y} \bigl({\mathbf{v}}_{h}^{-}(x,y_{j+\frac{1}{2} },z,t), \\ & \hphantom{\mathbf{f}^{y}_{i,j+\frac{1}{2},k}=}{\mathbf{v} }_{h}^{+}(x,y_{j+\frac{1}{2}},z,t) \bigr)\, dz \,dx\, dt, \end{aligned}$$

13 $$\begin{aligned}& \mathbf{f}^{z}_{ij,k+\frac{1}{2}}=\frac{1}{\Delta t} \frac{1}{\Delta x_{i}}\frac {1}{\Delta y_{j}} \\ & \hphantom{\mathbf{f}^{z}_{ij,k+\frac{1}{2}}=}{}\times \int _{t^{n}}^{t^{n+1}} \int _{x_{i-\frac{1}{2}}}^{x_{i+\frac{1}{2}}} \int _{y_{j-\frac{1}{2} }}^{y_{j+\frac{1}{2}}} \tilde{\mathbf{ f}}^{z} \bigl({\mathbf{v}}_{h}^{-}(x,y,z_{k+\frac{1}{2} },t), \\ & \hphantom{\mathbf{f}^{z}_{ij,k+\frac{1}{2}}=}{\mathbf{v} }_{h}^{+}(x,y,z_{k+\frac{1}{2}},t) \bigr) \,dy\, dx\, dt \end{aligned}$$

and the space-time averaged source term

14 $$\begin{aligned} \bar{\mathbf{S}} _{ijk} =&\frac{1}{\Delta t} \frac{1}{\Delta x_{i}}\frac {1}{\Delta y_{j}}\frac{1}{\Delta z_{k}} \\ &{}\times \int _{t^{n}}^{t^{n+1}} \int _{x_{i-\frac{1}{2}}}^{x_{i+\frac{1}{2}}} \int _{y_{j-\frac{1}{2} }}^{y_{j+\frac{1}{2}}} \int _{z_{k-\frac{1}{2}}}^{z_{k+\frac{1}{2}}}\mathbf{ S} \bigl(\mathbf{v} _{h}(x,y, \\ &{}z,t) \bigr)\, dz\,dy\,dx\,dt . \end{aligned}$$

We emphasize that the terms ${\mathbf{v}}_{h}$ in Eqs. (11)-(14), as well as in the few equations below, are piecewise space-time polynomials of degree *M* in *primitive variables*, computed according to a suitable LSDG predictor based on the formulation (3), as we will discuss in Section 2.4. This marks a striking difference with respect to traditional ADER schemes, in which such polynomials are instead computed in conserved variables and are denoted as $\mathbf{q}_{h}$ (see, e.g. Hidalgo and Dumbser 2011). The integrals over the smooth part of the non-conservative terms in Eq. (9) yield the following contribution,

15 $$\begin{aligned} \bar{\mathbf{P}} _{ijk} =&\frac{1}{\Delta t}\frac{1}{\Delta x_{i}} \frac {1}{\Delta y_{j}}\frac{1}{\Delta z_{k}} \\ &{}\times\int _{t^{n}}^{t^{n+1}} \int _{x_{i-\frac{1}{2}}}^{x_{i+\frac{1}{2}}} \int _{y_{j-\frac{1}{2} }}^{y_{j+\frac{1}{2}}} \int _{z_{k-\frac{1}{2}}}^{z_{k+\frac{1}{2}}}\mathbf{B}({\mathbf{v}}_{h}) \\ &{}\times \mathbf{M} \nabla{\mathbf{v}}_{h} \,dz\,dy\,dx\,dt , \end{aligned}$$

while the *jumps* across the element boundaries are treated within the framework of path-conservative schemes (Parés and Castro 2004; Pares 2006; Muñoz and Parés 2007; Castro et al. 2006; Castro et al. 2008a; Castro et al. 2008b) based on the Dal Maso-Le Floch-Murat theory (Dal Maso et al. 1995) as

16 $$\begin{aligned}& {{D}}^{x}_{i+\frac{1}{2},j,k} = \frac{1}{\Delta t}\frac{1}{\Delta y_{j}} \frac{1}{\Delta z_{k}} \\ & \hphantom{{{D}}^{x}_{i+\frac{1}{2},j,k} =}{}\times\int _{t^{n}}^{t^{n+1}} \int _{y_{j-\frac{1}{2}}}^{y_{j+\frac{1}{2}}} \int _{z_{k-\frac{1}{2} }}^{z_{k+\frac{1}{2}}} {\mathcal {D}}_{x} \bigl({ \mathbf{v}}_{h}^{-}(x_{i+\frac{1}{2}},y,z,t), \\ & \hphantom{{{D}}^{x}_{i+\frac{1}{2},j,k} =}{} {\mathbf {v}}_{h}^{+}(x_{i+\frac{1}{2} },y,z,t) \bigr) \,dz \, dy \, dt, \end{aligned}$$

17 $$\begin{aligned}& {{D}}^{y}_{i,j+\frac{1}{2},k} = \frac{1}{\Delta t}\frac{1}{\Delta x_{i}} \frac{1}{\Delta z_{k}} \\& \hphantom{{{D}}^{y}_{i,j+\frac{1}{2},k} =}{}\times \int _{t^{n}}^{t^{n+1}} \int _{x_{i-\frac{1}{2}}}^{x_{i+\frac{1}{2}}} \int _{z_{k-\frac{1}{2} }}^{z_{k+\frac{1}{2}}} {\mathcal {D}}_{y} \bigl({ \mathbf{v}}_{h}^{-}(x,y_{j+\frac{1}{2}},z,t), \\& \hphantom{{{D}}^{y}_{i,j+\frac{1}{2},k} =}{}{\mathbf {v}}_{h}^{+}(x,y_{j+\frac{1}{2} },z,t) \bigr) \,dz \, dx \, dt, \end{aligned}$$

18 $$\begin{aligned}& {{D}}^{z}_{i,j,k+\frac{1}{2}} = \frac{1}{\Delta t}\frac{1}{\Delta x_{i}} \frac{1}{\Delta y_{j}} \\& \hphantom{{{D}}^{y}_{i,j+\frac{1}{2},k} =}{}\times\int _{t^{n}}^{t^{n+1}} \int _{x_{i-\frac{1}{2}}}^{x_{i+\frac{1}{2}}} \int _{y_{j-\frac{1}{2} }}^{y_{j+\frac{1}{2}}} {\mathcal {D}}_{z} \bigl({ \mathbf{v}}_{h}^{-}(x,y,z_{k+\frac{1}{2}},t), \\& \hphantom{{{D}}^{y}_{i,j+\frac{1}{2},k} =}{}{\mathbf {v}}_{h}^{+}(x,y,z_{k+\frac{1}{2} },t) \bigr)\, dy \, dx \, dt . \end{aligned}$$

According to this approach, the following path integrals must be prescribed

19 $$\begin{aligned}& {\mathcal {D}}_{i}\bigl({\mathbf{v}}_{h}^{-},{ \mathbf{v}}_{h}^{+}\bigr) = \int_{0}^{1}\mathbf{B}_{i} \bigl(\Psi \bigl({\mathbf{v} }_{h}^{-},{\mathbf{v}}_{h}^{+},s\bigr) \bigr) \mathbf{M} \bigl(\Psi\bigl({\mathbf {v}}_{h}^{-},{\mathbf{v}}_{h}^{+},s \bigr) \bigr) \frac {\partial\Psi}{\partial s}\,ds, \\& \quad i \in \{ x,y,z \}, \end{aligned}$$

where $\Psi(s)$ is a path joining the left and right boundary extrapolated states ${\mathbf{v}}_{h}^{-}$ and ${\mathbf{v}}_{h}^{+}$ in state space of the primitive variables. The simplest option is to use a straight-line segment path

20 $$ \Psi= \Psi\bigl({\mathbf{v}}_{h}^{-},{ \mathbf{v}}_{h}^{+},s\bigr) ={\mathbf{v}}_{h}^{-} + s\bigl({ \mathbf{v}}_{h}^{+} - {\mathbf{v}}_{h}^{-}\bigr) ,\quad 0\leq s \leq1. $$

Pragmatic as it is,^2^ the choice of the path (20) allows to evaluate the terms ${\mathcal {D}}_{i}$ in (19) as

21 $$\begin{aligned} {\mathcal {D}}_{i}\bigl({\mathbf{v}}_{h}^{-},{ \mathbf{v}}_{h}^{+}\bigr) =& \biggl( \int_{0}^{1} \mathbf{B}_{i} \bigl(\Psi \bigl({\mathbf{v}}_{h}^{-},{\mathbf{v}}_{h}^{+},s\bigr) \bigr) \mathbf{M} \bigl(\Psi \bigl({\mathbf{v}}_{h}^{-},{\mathbf{v}}_{h}^{+},s \bigr) \bigr) \,ds \biggr) \\ &{}\times \bigl( {\mathbf{v}}_{h}^{+} - { \mathbf{v}}_{h}^{-} \bigr) , \end{aligned}$$

that we compute through a three-point Gauss-Legendre formula (Dumbser et al. 2010; Dumbser and Toro 2011a; Dumbser and Toro 2011b). The computation of the numerical fluxes $\tilde{\mathbf{ f}}^{i}$ in Eq. (11) requires the use of an approximate Riemann solver, see Toro (1999). In this work we have limited our attention to a local Lax-Friedrichs flux (Rusanov flux) and to the Osher-type flux proposed in Dumbser and Toro (2011b), Dumbser and Toro (2011a), Castro et al. (2015). Both of them can be written formally as

22 $$\begin{aligned}& \tilde{\mathbf{ f}}^{i} = \frac{1}{2} \bigl( \mathbf{f}^{i}\bigl(\mathbf{v}_{h}^{-}\bigr) + \mathbf {f}^{i}\bigl(\mathbf{v}_{h}^{+}\bigr) \bigr) - \frac{1}{2}\mathbf{D}_{i} \widetilde{\mathbf{M}} \bigl( \mathbf{v}_{h}^{+} - \mathbf{v}_{h}^{-} \bigr) , \\& \quad i \in \{ x,y,z \}, \end{aligned}$$

where $\mathbf{D}_{i} \geq0$ is a positive-definite dissipation matrix that depends on the chosen Riemann solver. For the Rusanov flux it simply reads

23 $$ \mathbf{D}^{\mathrm{Rusanov}}_{i} = |s_{\max}| \mathbf{I} , $$

where $|s_{\max}|$ is the maximum absolute value of the eigenvalues admitted by the PDE and **I** is the identity matrix. The matrix $\widetilde{\mathbf{M}}$ is a *Roe matrix* that allows to write the jumps in the conserved variables in terms of the jump in the primitive variables, i.e.

24 $$ \mathbf{q}_{h}^{+} - \mathbf{q}_{h}^{-} = \mathbf{Q}\bigl( \mathbf{v}_{h}^{+}\bigr) - \mathbf{Q}\bigl(\mathbf {v}_{h}^{-} \bigr) = \widetilde{\mathbf{M}} \bigl( \mathbf{v} _{h}^{+} - \mathbf{v}_{h}^{-} \bigr). $$

Since $\mathbf{M}= \partial\mathbf{Q}/ \partial\mathbf{V}$, the Roe matrix $\widetilde{\mathbf{M}}$ can be easily defined by a path integral as

25 $$\begin{aligned} \mathbf{Q}\bigl(\mathbf{v}_{h}^{+}\bigr) - \mathbf{Q}\bigl( \mathbf{v}_{h}^{-}\bigr) =& \int _{0}^{1} \mathbf{M} \bigl(\Psi\bigl( \mathbf{v}_{h}^{-},\mathbf{v}_{h}^{+},s\bigr)\bigr) \frac{\partial\Psi}{\partial s}\, ds \\ =& \widetilde{\mathbf{M}} \bigl( \mathbf{v}_{h}^{+} - \mathbf{v} _{h}^{-} \bigr), \end{aligned}$$

which in the case of the simple straight-line segment path (20) leads to the expression

26 $$ \widetilde{\mathbf{M}}= \int _{0}^{1} \mathbf{M}\bigl(\Psi\bigl( \mathbf{v}_{h}^{-},\mathbf {v}_{h}^{+},s\bigr)\bigr) \,ds. $$

In the case of the Osher-type flux, on the other hand, the dissipation matrix reads

27 $$ \mathbf{D}^{\mathrm{Osher}}_{i} = \int _{0}^{1} \bigl|\mathbf{A}_{i}\bigl(\Psi \bigl(\mathbf{v} _{h}^{-},\mathbf{v}_{h}^{+},s\bigr)\bigr)\bigr|\, ds , $$

with the usual definition of the matrix absolute value operator

28 $$ |\mathbf{A}|=\mathbf{R}|\boldsymbol{\Lambda}|\mathbf{R}^{-1} ,\quad | \boldsymbol{\Lambda }|=\operatorname{diag}\bigl(|\lambda_{1}|, | \lambda_{2}|, \ldots, |\lambda_{\nu}|\bigr) . $$

The path Ψ in Eqs. (27) and (26) is the same segment path adopted in (20) for the computation of the jumps ${\mathcal {D}}_{i}$.

### A novel WENO reconstruction in primitive variables

Since we want to compute the time averaged fluxes [cf. Eqs. (11)-(13)] and the space-time averaged sources [cf. Eq. (14)] directly from the primitive variables **V**, it is necessary to reconstruct a WENO polynomial in primitive variables. However, the underlying finite volume scheme (9) will still advance in time the cell averages of the conserved variables $\bar{\mathbf{Q}}_{ijk}^{n}$, which are the only known input quantities at the reference time level $t^{n}$. Hence, the whole procedure is performed through the following three simple steps:

1. We perform a *first* standard spatial WENO reconstruction of the conserved variables starting from the cell averages $\bar{\mathbf{Q} }_{ijk}^{n}$. This allows to obtain a reconstructed polynomial $\mathbf{w} _{h}(x,y,z,t^{n})$ in conserved variables valid within each cell.
2. Since $\mathbf{w}_{h}(x,y,z,t^{n})$ is defined at any point inside the cell, we simply *evaluate* it at the cell center in order to obtain the *point value* $\mathbf{Q}_{ijk}^{n}= \mathbf{w}_{h}(x_{i},y_{j},z_{k},t^{n})$. This conversion from cell averages $\bar{\mathbf{Q}}_{ijk}^{n}$ to point values $\mathbf{Q}_{ijk}^{n}$ is the **main key idea** of our new method, since the simple identity $\mathbf{Q}_{ijk}^{n} = \bar{\mathbf{Q}}_{ijk}^{n}$ is valid only up to second order of accuracy! After that, we perform a conversion from the point-values of the conserved variables to the point-values in primitive variables, i.e. we apply Eq. (5), thus obtaining the corresponding primitive variables $\mathbf{V}_{ijk}^{n} = \mathbf{V} (\mathbf{Q}_{ijk}^{n})$ at each cell center. This is the only step in the entire algorithm that needs a conversion from the conservative to the primitive variables.
3. Finally, from the point-values of the primitive variables at the cell centers, we perform a *second* WENO reconstruction to obtain a reconstruction polynomial in *primitive variables*, denoted as $\mathbf{p}_{h}(x,y,z,t^{n})$. This polynomial is then used as the initial condition for the new local space-time DG predictor in primitive variables described in Section 2.4.

As for the choice of the spatial WENO reconstruction, we have adopted a dimension-by-dimension reconstruction strategy, discussed in full details in our previous works (see Dumbser et al. 2013; Dumbser et al. 2014; Zanotti and Dumbser 2015). Briefly, we first introduce space-time reference coordinates $\xi,\eta,\zeta,\tau\in[0,1]$, defined by

29 $$ \begin{aligned} &x = x_{i-\frac{1}{2}} + \xi\Delta x_{i},\quad\quad y = y_{j-\frac{1}{2}} + \eta\Delta y_{j},\\ & z = z_{k-\frac{1}{2}} + \zeta \Delta z_{k},\quad\quad t = t^{n} + \tau\Delta t , \end{aligned} $$

and, along each spatial direction, we define a basis of polynomials $\{\psi_{l}(\lambda)\}_{l=1}^{M+1}$, each of degree *M*, formed by the $M+1$ Lagrange interpolating polynomials, that pass through the $M+1$ Gauss-Legendre quadrature nodes $\{\mu_{k}\}_{k=1}^{M+1}$. According to the WENO philosophy, a number of stencils is introduced such that the final polynomial is a data-dependent nonlinear combination of the polynomials computed from each stencil. Here, we use a fixed number $N_{s}$ of one-dimensional stencils, namely $N_{s}=3$ for odd order schemes (even polynomials of degree *M*), and $N_{s}=4$ for even order schemes (odd polynomials of degree *M*). For example, focusing on the *x* direction for convenience, every stencil along *x* is formed by the union of $M+1$ adjacent cells, i.e.

30 $$ \mathcal{S}_{ijk}^{s,x} = \bigcup _{e=i-L}^{i+R} {I_{ejk}}, $$

where $L=L(M,s)$ and $R=R(M,s)$ are the spatial extension of the stencil to the left and to the right.^3^

Now, an important difference emerges depending on whether we are reconstructing the conserved or the primitive variables. In the former case, corresponding to the computation of $\mathbf{w} _{h}(x,y,z,t^{n})$ at step 1 above, we require that the reconstructed polynomial must preserve the *cell-averages* of the *conserved variables* over each element $I_{ijk}$. Since the polynomials reconstructed along the *x* direction can be written as

31 $$ \mathbf{w}^{s,x}_{h}\bigl(x,t^{n} \bigr) = \sum_{r=0}^{M} \psi_{r}( \xi) \hat{\mathbf{w}} ^{n,s}_{ijk,r} := \psi_{r}(\xi) \hat{\mathbf{w}}^{n,s}_{ijk,r} , $$

the reconstruction equations read

32 $$\begin{aligned}& \frac{1}{\Delta x_{e}} \int _{x_{e-\frac{1}{2}}}^{x_{e+\frac{1}{2}}} \mathbf{w} _{h}^{x} \bigl(x,t^{n}\bigr) \,dx \\& \quad= \frac{1}{\Delta x_{e}} \int _{x_{e-\frac{1}{2} }}^{x_{e+\frac{1}{2}}} \psi_{r}\bigl(\xi(x) \bigr) \hat{\mathbf{w}}^{n,s}_{ijk,r} \, dx \\& \quad = {\bar{ \mathbf{Q}}}^{n}_{ejk}, \quad\forall{I}_{ejk} \in\mathcal {S}_{ijk}^{s,x} . \end{aligned}$$

Equations (32) provide a system of $M+1$ linear equations for the unknown coefficients $\hat{\mathbf{w}}^{n,s}_{ijk,r}$, which is conveniently solved through linear algebra packages. Once this operation has been performed for each stencil, we construct a data-dependent nonlinear combination of the resulting polynomials, i.e.

33 $$ \mathbf{w}_{h}^{x}\bigl(x,t^{n} \bigr) = \psi_{r}(\xi) \hat{\mathbf{w}}^{n}_{ijk,r},\quad \mbox{with } \hat{\mathbf{w}}^{n}_{ijk,r} = \sum _{s=1}^{N_{s}} \omega_{s} \hat{\mathbf{w}} ^{n,s}_{ijk,r} . $$

The nonlinear weights $\omega_{s}$ are computed according to the WENO approach (Jiang and Shu 1996) and their explicit expression can be found in Dumbser et al. (2013), Dumbser et al. (2014), Zanotti and Dumbser (2015). The whole procedure must be repeated along the two directions *y* and *z*. Hence, although each direction is treated separately, the net effect provides a genuine multidimensional reconstruction. We now proceed with the **key step** of the new algorithm presented in this paper and compute the *point values* of the conserved quantities at the cell centers, simply by *evaluating* the reconstruction polynomials in the barycenter of each control volume:

34 $$ \mathbf{Q}_{ijk}^{n} = \mathbf{w}_{h} \bigl( x_{i},y_{j},z_{k},t^{n} \bigr). $$

These point values of the conserved quantities $\mathbf{Q}_{ijk}^{n}$ are now converted into point values of the primitive variables $\mathbf{V}_{ijk}^{n}$, which requires only a single *cons-to-prim conversion* per cell. In RHD and RMHD, this is one of the most expensive and most delicate parts of the entire algorithm:

35 $$ \mathbf{V}_{ijk}^{n} = \mathbf{V} \bigl( \mathbf{Q}_{ijk}^{n} \bigr). $$

The reconstruction polynomials in primitive variables are spanned by the same basis functions $\psi_{r}(\xi)$ used for $\mathbf{w}_{h}$, hence

36 $$ \mathbf{p}^{s,x}_{h}\bigl(x,t^{n} \bigr) = \sum_{r=0}^{M} \psi_{r}( \xi) \hat{\mathbf{p}} ^{n,s}_{ijk,r} := \psi_{r}(\xi) \hat{\mathbf{p}}^{n,s}_{ijk,r} . $$

According to step 3 listed above, we now require that the reconstructed polynomial must interpolate the *point-values* of the *primitive variables* at the centers of the cells forming each stencil, i.e.

37 $$ \mathbf{p}_{h}^{x}\bigl(x_{e},t^{n} \bigr) = \psi_{r}\bigl(\xi(x_{e})\bigr) \hat{ \mathbf{p}}^{n,s}_{ijk,r} = \mathbf{V} _{ejk}^{n} ,\quad \forall{I}_{ejk} \in\mathcal{S}_{ijk}^{s,x}. $$

The reconstruction equations (37) will also generate a system of $M+1$ linear equations for the unknown coefficients $\hat{\mathbf{p}}^{n,s}_{ijk,r}$. The rest of the WENO logic applies in the same way, leading to

38 $$ \mathbf{p}_{h}^{x}\bigl(x,t^{n} \bigr) = \psi_{r}(\xi) \hat{\mathbf{p}}^{n}_{ijk,r}, \quad\mbox{with } \hat{\mathbf{p}}^{n}_{ijk,r} = \sum _{s=1}^{N_{s}} \omega_{s} \hat{\mathbf{p}} ^{n,s}_{ijk,r} . $$

We emphasize that thanks to our polynomial WENO reconstruction (instead of the original point-wise WENO reconstruction of Jiang and Shu 1996), the point-value of $\mathbf{w} _{h}(x,y,z,t^{n})$ at each cell center, which is required at step 2 above, is promptly available after evaluating the basis functions at the cell center. In other words, there is no need to perform any special transformation from cell averages to point-values via Taylor series expansions, like in Buchmüller and Helzel (2014), Buchmüller et al. (2015). On the other hand, since the WENO reconstruction is performed twice, once for the conserved variables and once for the primitive variables, we expect that our new approach will become convenient in terms of computational efficiency only for those systems of equations characterized by relations $\mathbf{V}(\mathbf{Q})$ that cannot be written in closed form. In such circumstances, in fact, reducing the number of *cons-to-prim conversions* from $M (M+1)^{d+1} + d (M+1)^{d}$ in *d* space dimensions (due to the space-time predictor and the numerical flux computation in the finite volume scheme) to just *one single conversion* per cell will compensate for the double WENO reconstruction in space that we must perform. On the contrary, for systems of equations, such as the compressible Euler, for which the *cons-to-prim conversion* is analytic, no benefit will be reported in terms of computational efficiency, but still a significant benefit will be reported in terms of numerical accuracy. All these comments will be made quantitative in Section 3.

### A local space-time DG predictor in primitive variables

#### Description of the predictor

As already remarked, the computation of the fluxes through the integrals (11)-(13) is more conveniently performed if the primitive variables are available at each space-time quadrature point. In such a case, in fact, no conversion from the conserved to the primitive variables is required. According to the discussion of the previous Section, it is possible to obtain a polynomial $\mathbf{p}_{h}(x,y,z,t^{n})$ in primitive variables at the reference time $t^{n}$. This is however not enough for a high accurate computation of the numerical fluxes, and $\mathbf{p}_{h}(x,y,z,t^{n})$ must be evolved in time, locally for each cell, in order to obtain a polynomial $\mathbf{v}_{h}(x,y,z,t)$ approximating the solution at any time in the range $[t^{n};t^{n+1}]$.

To this extent, we need an operation, to be performed locally for each cell, which uses as input the high order polynomial $\mathbf{v}_{h}$ obtained from the WENO reconstruction, and gives as output its evolution in time, namely

39 $$ \mathbf{p}_{h}\bigl(x,y,z,t^{n}\bigr) \xrightarrow{\mathit{LSDG}} \mathbf{v}_{h}(x,y,z,t) , \quad t\in \bigl[t^{n};t^{n+1} \bigr] . $$

This can be obtained through an element-local space-time discontinuous Galerkin predictor that is based on the *weak* integral form of Eq. (3). From a mathematical point of view, Eq. (3) is a hyperbolic system in non-conservative form. Therefore, the implementation of the space-time discontinuous Galerkin predictor follows strictly the strategy already outlined in Dumbser et al. (2014) for non-conservative systems. Here we recall briefly the main ideas, focusing on the novel aspects implied by the formulation of Eq. (3). The sought polynomial $\mathbf{v}_{h}(x,y,z,t)$ is supposed to be expanded in space and time as

40 $$ \mathbf{v}_{h} = \mathbf{v}_{h}(\boldsymbol{\xi},\tau) = \theta _{l} (\boldsymbol{\xi},\tau ) \hat{\mathbf{v}}^{n}_{l} , $$

where the degrees of freedom $\hat{\mathbf{v}}^{n}_{l}$ are the unknowns. The space-time basis functions $\theta_{l}$ are given by a dyadic product of the Lagrange interpolation polynomials that pass through the Gauss-Legendre quadrature points, i.e. the tensor-product quadrature points on the hypercube $[0,1]^{d+1}$, see Stroud (1971). The system (3) is first rephrased in terms of the reference coordinates *τ* and $\boldsymbol{\xi} = (\xi,\eta ,\zeta)$, yielding

41 $$ \frac{\partial{\mathbf{V}}}{\partial\tau} + \mathbf{C}_{1}^{\ast}\frac{\partial{\mathbf{V}}}{\partial\xi} + \mathbf {C}_{2}^{\ast}\frac{\partial{\mathbf{V}}}{\partial\eta} + \mathbf{C}_{3}^{\ast}\frac {\partial{\mathbf{V}}}{\partial\zeta} =\mathbf{S}^{\ast}, $$

with

42 $$ \begin{aligned} &\mathbf{C}_{1}^{\ast}= \frac{\Delta t}{\Delta x_{i}} \mathbf{C}_{1},\quad\quad \mathbf{C}_{2}^{\ast}= \frac{\Delta t}{\Delta y_{j}} \mathbf{C}_{2}, \\ & \mathbf{C}_{3}^{\ast}= \frac{\Delta t}{\Delta z_{k}} \mathbf{C}_{3},\quad\quad \mathbf{ S}^{\ast}= \Delta t \mathbf{M}^{-1}\mathbf{ S}. \end{aligned} $$

Expression (41) is then multiplied by the piecewise space-time polynomials $\theta_{k}(\xi,\eta,\zeta,\tau)$ and integrated over the space-time reference control volume, thus providing

43 $$\begin{aligned}& \int _{0}^{1} \int _{0}^{1} \int _{0}^{1} \int _{0}^{1} \theta_{k} \frac{\partial{\mathbf{v}_{h}}}{\partial\tau}\, d \boldsymbol{\xi}\, d\tau \\& \quad= \int _{0}^{1} \int _{0}^{1} \int _{0}^{1} \int _{0}^{1} \theta_{k} \biggl( \mathbf{S}^{\ast}- \mathbf{C}_{1}^{\ast}\frac{\partial \mathbf{v} _{h}}{\partial\xi} \\& \quad\quad{}- \mathbf{C}_{2}^{\ast}\frac{\partial\mathbf {v}_{h}}{\partial\eta } - \mathbf{C}_{3}^{\ast}\frac{\partial\mathbf{v}_{h}}{\partial\zeta} \biggr)\, d \boldsymbol{ \xi}\, d\tau , \end{aligned}$$

where we have replaced **V** with its discrete representation $\mathbf{v}_{h}$. Integrating the first term by parts in time yields

44 $$\begin{aligned}& \int _{0}^{1} \int _{0}^{1} \int _{0}^{1} \theta_{k}(\boldsymbol{ \xi},1) \mathbf{v}_{h}(\boldsymbol{\xi},1) \, d \boldsymbol{\xi} \\& \quad\quad{}- \int _{0}^{1} \int _{0}^{1} \int _{0}^{1} \int _{0}^{1} \biggl(\frac{\partial}{\partial\tau} \theta_{k} \biggr) \mathbf{v} _{h}(\boldsymbol{\xi},\tau) \, d \boldsymbol{\xi}\, d\tau \\& \quad= \int _{0}^{1} \int _{0}^{1} \int _{0}^{1} \theta _{k}(\boldsymbol{ \xi},0) \mathbf{p}_{h}\bigl(\boldsymbol{\xi},t^{n}\bigr) \, d \boldsymbol {\xi} \\& \quad\quad{}+ \int _{0}^{1} \int _{0}^{1} \int _{0}^{1} \int _{0}^{1} \theta_{k} \biggl( \mathbf{S}^{\ast}- \mathbf{C}_{1}^{\ast}\frac{\partial \mathbf{v} _{h}}{\partial\xi} \\& \quad\quad{}- \mathbf{C}_{2}^{\ast}\frac{\partial\mathbf {v}_{h}}{\partial\eta } - \mathbf{C}_{3}^{\ast}\frac{\partial\mathbf{v}_{h}}{\partial\zeta} \biggr) \, d \boldsymbol{ \xi}\, d\tau . \end{aligned}$$

Equation (44) is an element-local nonlinear algebraic equation that must be solved locally for each grid-cell in the unknowns $\hat{\mathbf{v}}^{n}_{l}$. In practice, we solve the system of equations (44) through a discrete Picard iteration, see Dumbser and Zanotti (2009), Hidalgo and Dumbser (2011), where additional comments about its solution can be found.

#### An efficient initial guess for the predictor

A proper choice of the initial guess for each of the space-time degrees of freedom $\hat{\mathbf{v}}_{l}$ can improve the convergence of the Picard process. The easiest strategy is to set $\mathbf{v}_{h}(\mathbf{x},t) = \mathbf{p} _{h}(\mathbf {x},t^{n})$ i.e. the reconstruction polynomial is simply extended as a constant in time. This is, however, not the best approach. A better strategy for obtaining a good initial guess for the LSDG predictor was presented in Hidalgo and Dumbser (2011), and it is based on the implementation of a MUSCL scheme for the explicit terms, plus a second-order Crank-Nicholson scheme in case stiff source terms are present. In the following, we refer to this version of the initial guess for the LSDG predictor as the MUSCL-CN initial guess. If the source terms are not stiff, however, an even more efficient approach is possible which is based on a space-time extension of multi-level Adams-Bashforth-type ODE integrators. For that purpose, the space-time polynomial denoted by $\mathbf{v}^{n-1}_{h}(\mathbf{x},t)$ obtained during the previous time step $[t^{n-1},t^{n}]$ is simply *extrapolated in time* to the new time step $[t^{n},t^{n+1}]$ by simple L2 projection:

45 $$\begin{aligned}& \int _{I_{ijk}} \int _{t^{n}}^{t^{n+1}} \theta_{k}(\mathbf {x},t) \mathbf{v}^{n}_{h}(\mathbf{x},t) \, dt \, d\mathbf{x} \\& \quad = \int _{I_{ijk}} \int _{t^{n}}^{t^{n+1}} \theta_{k}(\mathbf {x},t) \mathbf{v}^{n-1}_{h}(\mathbf{x},t) \, dt \, d\mathbf{x}. \end{aligned}$$

In terms of the degrees of freedom $\hat{\mathbf{v}}^{n}_{l}$ and $\hat {\mathbf{v}}^{n-1}_{l}$ this relation becomes

46 $$\begin{aligned}& \int _{0}^{1} \int _{0}^{1} \int _{0}^{1} \int _{0}^{1} \theta_{k}(\boldsymbol{ \xi},\tau) \theta_{l}(\boldsymbol{\xi},\tau) \hat{\mathbf{v}} ^{n}_{l} \, dt \, d\boldsymbol{\xi} \\& \quad = \int _{0}^{1} \int _{0}^{1} \int _{0}^{1} \int _{0}^{1} \theta_{k}(\boldsymbol{ \xi},\tau) \theta_{l}\bigl(\boldsymbol{\xi},\tau '\bigr) \hat{\mathbf{v}} ^{n-1}_{l} \, dt \, d\boldsymbol{\xi}, \end{aligned}$$

with $\tau' = 1 + \tau\frac{\Delta t^{n}}{\Delta t^{n-1}}$ and $\Delta t^{n-1} = t^{n} - t^{n-1}$.

In the following, we refer to this second version of the initial guess for the LSDG predictor as the Adams-Bashforth (AB) initial guess. In Table 1 we show a comparison among the performances of the LSDG predictor with these two different implementations of the initial guess.

**Table 1:** **CPU time comparison among different versions of the initial guesses for the LSDG predictor**

	**MUSCL-CN**	**Adams-Bashforth**
$\mathbb{P}_{0}\mathbb{P}_{2}$	1.0	0.64
$\mathbb{P}_{0}\mathbb{P}_{3}$	1.0	0.75
$\mathbb{P}_{0}\mathbb{P}_{4}$	1.0	0.72

The comparison has been performed for the isentropic vortex solution and the numbers have been normalized to the value obtained with the traditional MUSCL-CN initial guess (see Section 2.4.2 for more details).

## Numerical tests with the new ADER-WENO finite volume scheme in primitive variables

In the following we explore the properties of the new ADER-WENO finite volume scheme by solving a wide set of test problems belonging to four different systems of equations: the classical Euler equations, the relativistic hydrodynamics (RHD) and magnetohydrodynamics (RMHD) equations and the Baer-Nunziato equations for compressible two-phase flows. For the sake of clarity, we introduce the notation ‘ADER-Prim’ to refer to the novel approach of this work for which both the spatial WENO reconstruction and the subsequent LSDG predictor are performed on the primitive variables. On the contrary, we denote the traditional ADER implementation, for which both the spatial WENO reconstruction and the LSDG predictor are performed on the conserved variables, as ‘ADER-Cons’. In a few circumstances, we have also compared with the ‘ADER-Char’ scheme, namely a traditional ADER scheme in which, however, the spatial reconstruction is performed on the characteristic variables. In this Section we focus our attention on finite volume schemes, which, according to the notation introduced in Dumbser et al. (2008a), are denoted as $\mathbb{P}_{0}\mathbb{P}_{M}$ methods, where *M* is the degree of the approximating polynomial. In Section 4 a brief account is given to discontinuous Galerkin methods, referred to as $\mathbb{P}_{N}\mathbb{P}_{N}$ methods, for which an ADER-Prim version is also possible.

### Euler equations

First of all we consider the solution of the classical Euler equations of compressible gas dynamics, for which the vectors of the conserved variables **Q** and of the fluxes $\mathbf{f}^{x}$, $\mathbf{f}^{y}$ and $\mathbf{f}^{z}$ are given respectively by

47 $$ \begin{aligned} &\mathbf{Q} =\begin{pmatrix} \rho\\ \rho v_{x} \\ \rho v_{y} \\ \rho v_{z} \\ E \end{pmatrix} , \quad\quad\mathbf{f}^{x}= \begin{pmatrix} \rho v_{x} \\ \rho v_{x}^{2} + p \\ \rho v_{x}v_{y} \\ \rho v_{x}v_{z} \\ v_{x}(E+p) \end{pmatrix} , \\ &\mathbf{f}^{y}= \begin{pmatrix} \rho v_{y} \\ \rho v_{x}v_{y} \\ \rho v_{y}^{2} + p \\ \rho v_{y}v_{z} \\ v_{y}(E+p) \end{pmatrix} ,\quad\quad \mathbf{f}^{z}= \begin{pmatrix} \rho v_{z} \\ \rho v_{x}v_{z} \\ \rho v_{y}v_{z} \\ \rho v_{z}^{2} + p \\ v_{z}(E+p) \end{pmatrix} . \end{aligned} $$

Here $v_{x}$, $v_{y}$ and $v_{z}$ are the velocity components, *p* is the pressure, *ρ* is the mass density, $E=p/(\gamma-1)+\rho(v_{x}^{2}+v_{y}^{2}+v_{z}^{2})/2$ is the total energy density, while *γ* is the adiabatic index of the supposed ideal gas equation of state, which is of the kind $p=\rho\epsilon(\gamma-1)$, *ϵ* being the specific internal energy.

#### 2D isentropic vortex

It is important to assess the convergence properties of the new scheme, in particular comparing with the traditional ADER scheme in conserved and in characteristic variables. To this extent, we have studied the two-dimensional isentropic vortex, see e.g. Hu and Shu (1999). The initial conditions are given by a uniform mean flow, to which a perturbation is added, such that

48 $$ ( \rho,v_{x},v_{y},v_{z},p ) =(1+\delta\rho, 1+ \delta v_{x}, 1+\delta v_{y}, 0, 1+\delta p) , $$

with

49 $$ \begin{pmatrix} \delta\rho\\ \delta v_{x} \\ \delta v_{y} \\ \delta p \end{pmatrix} = \begin{pmatrix} (1+\delta T)^{1/(\gamma-1)}-1 \\ -(y-5)\epsilon/2\pi\exp{[0.5(1-r^{2})]} \\ (x-5)\epsilon/2\pi\exp{[0.5(1-r^{2})]} \\ (1+\delta T)^{\gamma/(\gamma-1)}-1 \end{pmatrix}. $$

Whatever the perturbation *δT* in the temperature is, it is easy to verify that there is not any variation in the specific entropy $s=p/\rho^{\gamma}$, and the flow is advected smoothly and isentropically with velocity $v=(1,1,0)$. We have solved this test over the computational domain $\Omega =[0;10]\times [0;10]$, assuming

50 $$ \delta T=-\frac{\epsilon^{2}(\gamma-1)}{8\gamma\pi^{2}} \exp {\bigl(1-r^{2}\bigr)} , $$

with $r^{2}=(x-5)^{2}+(y-5)^{2}$, vortex strength $\epsilon=5$ and adiabatic index $\gamma=1.4$. Table 2 contains the results of our calculation, in which we have compared the convergence properties of three different finite volume ADER schemes: ADER-Prim, ADER-Cons and ADER-Char, obtained with the Osher-type Riemann solver, see Dumbser and Toro (2011b). While all the schemes converge to the nominal order, it is interesting to note that the smallest $L_{2}$ error is obtained for the *new* ADER finite volume scheme in *primitive variables*, and that the difference with respect to the other two reconstructions increases with the order of the method.

**Table 2:** $\pmb{L_{2}}$ **errors of the mass density and corresponding convergence rates for the 2D isentropic vortex problem**

**2D isentropic vortex problem**								
	$\boldsymbol{N}_{\boldsymbol{x}}$	**ADER-Prim**		**ADER-Cons**		**ADER-Char**		**Theor.**
		$\boldsymbol{L}_{\boldsymbol{2}}$ **error**	$\boldsymbol{L}_{\boldsymbol{2}}$ **order**	$\boldsymbol{L}_{\boldsymbol{2}}$ **error**	$\boldsymbol{L}_{\boldsymbol{2}}$ **order**	$\boldsymbol{L}_{\boldsymbol{2}}$ **error**	$\boldsymbol{L}_{\boldsymbol{2}}$ **order**	
$\mathbb{P}_{0}\mathbb{P}_{2}$	100	4.060E-03	-	5.028E-03	-	5.010E-03	-	3
	120	2.359E-03	2.98	2.974E-03	2.88	2.968E-03	2.87	
	140	1.489E-03	2.98	1.897E-03	2.92	1.893E-03	2.92	
	160	9.985E-04	2.99	1.281E-03	2.94	1.279E-03	2.94	
	200	5.118E-04	2.99	6.612E-04	2.96	6.607E-04	2.96	
$\mathbb{P}_{0}\mathbb{P}_{3}$	50	2.173E-03	-	4.427E-03	-	5.217E-03	-	4
	60	8.831E-04	4.93	1.721E-03	5.18	2.232E-03	4.65	
	70	4.177E-04	4.85	8.138E-04	4.85	1.082E-03	4.69	
	80	2.194E-04	4.82	4.418E-04	4.57	5.746E-04	4.74	
	100	7.537E-05	4.79	1.605E-04	4.53	1.938E-04	4.87	
$\mathbb{P}_{0}\mathbb{P}_{4}$	50	2.165E-03	-	3.438E-03	-	3.416E-03	-	5
	60	6.944E-04	6.23	1.507E-03	4.52	1.559E-03	4.30	
	70	3.292E-04	4.84	7.615E-04	4.43	7.615E-04	4.65	
	80	1.724E-04	4.84	4.149E-04	4.55	4.148E-04	4.55	
	100	5.884E-05	4.82	1.449E-04	4.71	1.448E-04	4.72	

A comparison is shown among the reconstruction in primitive variables (ADER-Prim), in conserved variables (ADER-Cons) and in characteristic variables (ADER-Char). The Osher-type numerical flux has been used.

In addition to the convergence properties, we have compared the performances of the Adams-Bashforth version of the initial guess for the LSDG predictor with the traditional version based on the MUSCL-CN algorithm. The comparison has been performed over a $100\times100$ uniform grid. The results are shown in Table 1, from which we conclude that the Adams-Bashforth initial guess is indeed computationally more efficient in terms of CPU time. However, we have also experienced that it is typically less robust, and in some of the most challenging numerical tests discussed in the rest of the paper we had to use the more traditional MUSCL-CN initial guess.

#### Sod’s Riemann problem

**Figure 1:**
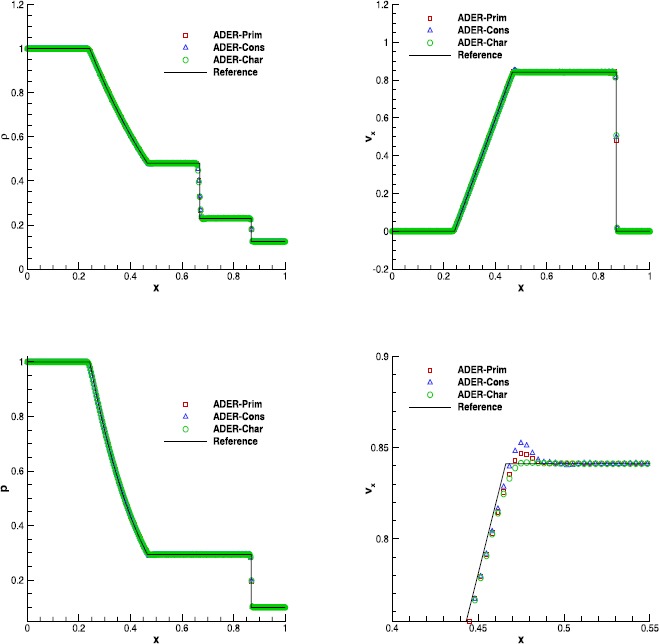
**Solution of Sod’s Riemann problem with the fourth order ADER-WENO scheme at time** $\pmb{t=0.2}$ **.** The bottom right panel shows a magnification of the velocity at the tail of the rarefaction.

We have then solved the classical Riemann problem named after Sod (Sod 1978), assuming an adiabatic index $\gamma=1.4$, and evolved until $t_{\mathrm{final}}=0.2$. In spite of the fact that this is a one-dimensional test, we have evolved this problem in two spatial dimensions over the domain $[0,1]\times[-0.2,0.2]$, using periodic boundary conditions along the passive *y* direction. In Figure 1 we show the comparison among the solutions obtained with ADER-Prim, ADER-Cons and ADER-Char, together with the exact solution provided in Toro (1999). We have adopted the finite volume scheme at the fourth order of accuracy, namely the $\mathbb{P}_{0}\mathbb{P}_{3}$ scheme, in combination with the Rusanov numerical flux and using 400 cells along the *x*-direction. Although all of the ADER implementations show a very good agreement with the exact solution, a closer look at the tail of the rarefaction, highlighted in the bottom right panel, reveals that the ADER-Cons scheme is actually the worst one, while the solution obtained with ADER-Prim is more similar to the reconstruction in characteristic variables. On the contrary, in terms of CPU-time, ADER-Prim is not convenient for this system of equations because the price paid for performing the double WENO reconstruction in space is not significantly compensated by the reduced number of conversions that are needed from the conserved to the primitive variables.Table 3 reports the CPU times, normalized with respect to the ADER-Prim implementation, for different orders of accuracy, showing that the ADER-Prim scheme is ∼25 % slower than the traditional ADER-Cons scheme. As we will see in Table 5 of Section 3.2, the comparison will change in favor of ADER-Prim schemes, when the relativistic equations are solved instead.

**Table 3:** **CPU time comparison among different ADER implementations for the Sod Riemann problem**

	**ADER-Prim**	**ADER-Cons**	**ADER-Char**
$\mathbb{P}_{0}\mathbb{P}_{2}$	1.0	0.74	0.81
$\mathbb{P}_{0}\mathbb{P}_{3}$	1.0	0.74	0.80
$\mathbb{P}_{0}\mathbb{P}_{4}$	1.0	0.77	0.81

The numbers have been normalized to the value obtained with ADER-Prim.

#### Interacting blast waves

**Figure 2:**
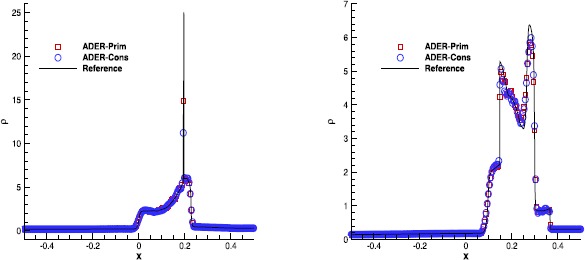
**Solution of the interacting Blast-Wave problem at time** $\pmb{t=0.028}$ **(left panel) and at time** $\pmb{t=0.038}$ **(right panel) obtained with the fourth order ADER-WENO scheme.** The computation has been performed over a uniform grid of 500 cells.

The interaction between two blast waves was first proposed by Woodward and Colella (1984) and it is now a standard test for computational fluid dynamics. The initial conditions are given by

51 $$ (\rho,v_{x},p)= \textstyle\begin{cases} (1.0,0.0,10^{3}) &\mbox{if } -0.5 < x < -0.4 , \\ (1.0,0.0,10^{-2}) &\mbox{if } -0.4 < x < 0.4 , \\ (1.0,0.0,10^{2}) &\mbox{if } 0.4 < x < 0.5 , \end{cases} $$

where the adiabatic index is $\gamma=1.4$. We have evolved this problem in two spatial dimensions over the domain $[-0.6,0.6]\times[-0.5,0.5]$, using reflecting boundary conditions in *x* direction and periodic boundary conditions along the *y* direction. The results of our calculations, obtained with the $\mathbb{P}_{0}\mathbb{P}_{3}$ scheme, are reported in Figure 2, where only the one-dimensional cuts are shown. The number of cells chosen along the *x*-direction, namely $N_{x}=500$, is not particularly large, at least for this kind of challenging problem. This has been intentionally done to better highlight potential differences among the two alternative ADER-Prim and ADER-Cons schemes. As it turns out from the figure, the two methods are very similar in terms of accuracy: the sharp peak in the density at time $t=0.028$ (left panel) is somewhat better resolved through the ADER-Prim, while the opposite is true for the highest peak at time $t=0.038$ (right panel). On the overall, however, the two schemes perform equally well for this test.

#### Double Mach reflection problem

**Figure 3:**
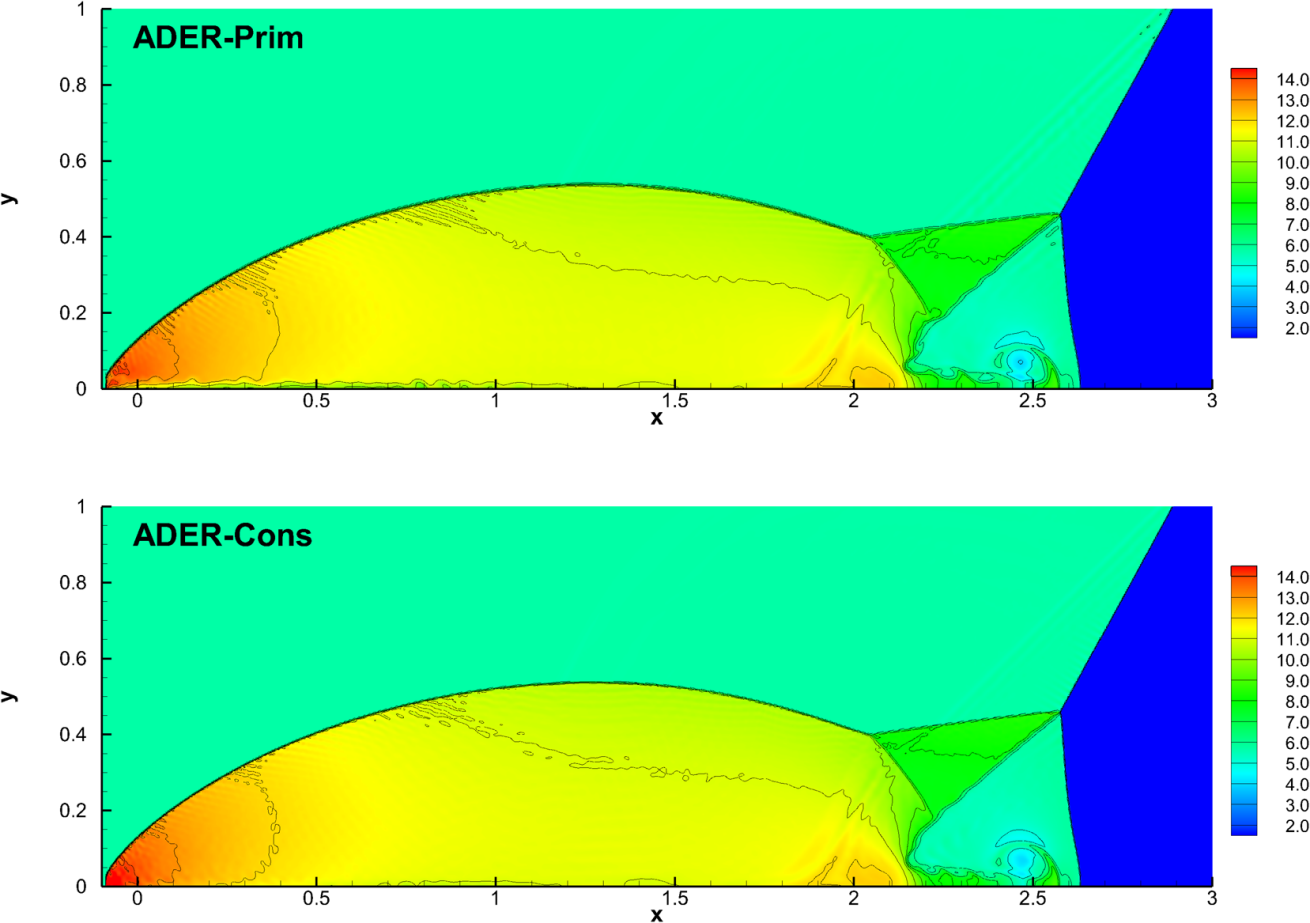
**Double Mach reflection problem at time** $\pmb{t=0.2}$ **obtained with the fourth order ADER-WENO scheme and the Rusanov Riemann solver. The computation has been performed over a uniform grid of** $\pmb{1\mbox{,}200\times 300}$ **cells.** Top panel: mass density distribution obtained with ADER-Prim. Bottom panel: mass density distribution obtained with ADER-Cons.

As a representative test for the Euler equations in two space dimensions, we have considered the *double Mach reflection problem*, which implies the interaction of several waves. The dynamics of this problem is triggered by a shock wave propagating towards the right with a Mach number $M=10$, and intersecting the *x*-axis at $x=1/6$ with an inclination angle of $\alpha=60^{\circ}$. The initial states ahead and behind the shock are fixed after solving the Rankine-Hugoniot conditions, obtaining

52 $$\begin{aligned}& (\rho, u, v, p) ( \mathbf{x},t=0) \\& \quad= \textstyle\begin{cases} \frac{1}{\gamma}(8.0, 8.25, 0.0, 116.5), &\text{if } x'< 0.1, \\ (1.0, 0.0, 0.0, \frac{1}{\gamma}), &\text{if } x'\geq0.1, \end{cases}\displaystyle \end{aligned}$$

where $x' = (x - 1/6) \cos\alpha- y \sin\alpha$. The adiabatic index is $\gamma=1.4$. We fix inflow and outflow boundary conditions on the left side and on the right of the numerical domain, respectively, while on the bottom we have used reflecting boundary conditions. At the top we must impose the exact solution of an isolated moving oblique shock wave with the same shock Mach number $M_{s}=10$. We have solved the test over the rectangle $\Omega= [0;3.0] \times[0;1]$, covered by a uniform grid composed of $1\mbox{,}200\times300$ cells, using the Rusanov Riemann solver and a fourth order finite volume scheme. The two panels of Figure 3 show the comparison of the solution at time $t=0.2$ obtained with the ADER-Prim (top panel) and with the ADER-Cons (bottom panel) scheme. The results are very similar in the two cases.

As a tentative conclusion about the performances of ADER-Prim for the Euler equations, we may say that, although it is the most accurate on smooth solutions (see Table 2), and comparable to a traditional ADER with reconstruction in characteristic variables, it is computationally more expensive than ADER-Cons and ADER-Char. Hence, ADER-Prim will rarely become the preferred choice in standard applications for the Euler equations.

### Relativistic hydrodynamics and magnetohydrodynamics

From a formal point of view, the equations of special relativistic hydrodynamics and magnetohydrodynamics can be written in conservative form like the classical Euler equations (see, however, the comments below), namely as in Eq. (1), with the vectors of the conserved variables and of the corresponding fluxes given by

53 $$ \mathbf{Q} =\begin{bmatrix} D \\ S_{j} \\ U \\ B^{j} \end{bmatrix},\quad\quad \mathbf{f}^{i}=\begin{bmatrix} v^{i} D \\ W^{i}_{j} \\ S^{i} \\ \epsilon^{jik}E^{k} \end{bmatrix} , \quad i=x,y,z , $$

where the conserved variables $(D,S_{j},U,B_{j})$ can be expressed as^4^

54 $$\begin{aligned}& D = \rho W , \end{aligned}$$

55 $$\begin{aligned}& S_{i} = \rho h W^{2} v_{i} + \epsilon_{ijk}E_{j} B_{k}, \end{aligned}$$

56 $$\begin{aligned}& U = \rho h W^{2} - p + \frac{1}{2} \bigl(E^{2} + B^{2}\bigr) , \end{aligned}$$

while the spatial projection of the energy-momentum tensor of the fluid is (Del Zanna et al. 2007)

57 $$\begin{aligned} W_{ij} \equiv&\rho h W^{2} v_{i} v_{j} - E_{i} E_{j} - B_{i} B_{j} \\ &{}+ \biggl[p + \frac {1}{2}\bigl(E^{2}+B^{2}\bigr) \biggr] \delta_{ij} . \end{aligned}$$

Here $\epsilon_{ijk}$ is the Levi-Civita tensor and $\delta_{ij}$ is the Kronecker symbol. We have used the symbol $h=1+\epsilon+p/\rho$ to denote the specific enthalpy of the plasma and in all our calculations the usual ideal gas equation of state has been assumed.

The components of the electric and of the magnetic field in the laboratory frame are denoted by $E_{i}$ and $B_{i}$, while the Lorentz factor of the fluid with respect to this reference frame is $W=(1-v^{2})^{-1/2}$. We emphasize that the electric field does not need to be evolved in time under the assumption of infinite electrical conductivity, since it can always be computed in terms of the velocity and of the magnetic field as $\vec{E} = - \vec{v} \times\vec{B}$.

Although formally very similar to the classical gas dynamics equations, their relativistic counterpart present two fundamental differences. The first one is that, while the physical fluxes $\mathbf{f}^{i}$ of the classical gas dynamics equations can be written analytically in terms of the conserved variables, i.e. $\mathbf{f}^{i}=\mathbf{f}^{i}(\mathbf{Q})$, those of the relativistic hydrodynamics (or magnetohydrodynamics) equations need the knowledge of the primitive variables, i.e. $\mathbf{f}^{i}=\mathbf{f}^{i}(\mathbf{V})$ for RMHD. The second difference is that, in the relativistic case, the conversion from the conserved to the primitive variables, i.e. the operation $(D,S_{j},U,B_{j})\rightarrow(\rho,v_{i},p,B_{i})$, is not analytic, and it must be performed numerically through some appropriate iterative procedure. Since in an ADER scheme such a conversion must be performed in each space-time degree of freedom of the space-time DG predictor and at each Gaussian quadrature point for the computation of the fluxes in the finite volume scheme, we may expect a significant computational advantage by performing the WENO reconstruction and the LSDG predictor directly on the primitive variables. In this way, in fact, the conversion $(D,S_{j},U,B_{j})\rightarrow(\rho,v_{i},p,B_{i})$ is required only once at the cell center (see Section 2.3), and not in each space-time degree of freedom of the predictor and at each Gaussian point for the quadrature of the numerical fluxes. We emphasize that the choice of the variables to reconstruct for the relativistic velocity is still a matter of debate. The velocity $v_{i}$ may seem the most natural one, but, as first noticed by Komissarov (1999), reconstructing $W v_{i}$ can increase the robustness of the scheme. However, this is not always the case (see Section 3.2.5 below) and in our tests we have favored either the first or the second choice according to convenience. Concerning the specific strategy adopted to recover the primitive variables, in our numerical code we have used the third method reported in Section 3.2 of Del Zanna et al. (2007). Alternative methods can be found in Noble et al. (2006), Rezzolla and Zanotti (2013).

Finally, there is an important formal change in the transition from purely hydrodynamics systems to genuinely magnetohydrodynamics systems. As already noticed by Londrillo and Del Zanna (2000), the RMHD equations should not be regarded as a mere extension of the RHD ones, with just a larger number of variables to evolve. Rather, their formal structure is better described in terms of a coupled system of conservation laws (the five equations for the dynamics of the plasma) and a set of Hamilton-Jacobi equations, those for the evolution of the vector potential of the magnetic field (Jin and Xin 1998). The different mathematical structure of the RMHD equations reflects the existence of the divergence-free property of the magnetic field, which must be ensured at all times during the evolution. Numerically, we have adopted a simplified and well known approach, which consists of augmenting the system (1) with an additional equation for a scalar field Φ, aimed at propagating away the deviations from $\vec{\nabla}\cdot\vec{B}=0$. We therefore need to solve

58 $$ \partial_{t} \Phi+ \partial_{i} B^{i} = -\kappa\Phi , $$

while the fluxes for the evolution of the magnetic field are also changed, namely $\mathbf{f}^{i}(B^{j})\rightarrow\epsilon^{jik}E^{k} + \Phi \delta^{ij}$, where $\kappa\in[1;10]$ in most of our calculations. Originally introduced by Dedner et al. (2002) for the classical MHD equations, this approach has been extended to the relativistic regime by Palenzuela et al. (2009). More information about the mathematical structure of the RMHD equations can be found in Anile (1990), Balsara (2001), Komissarov (1999), Del Zanna et al. (2007), Antón et al. (2010).

In the following, we first limit our attention to a few physical systems for which $B_{i}=E_{i}=0$, hence to relativistic hydrodynamics, and then we consider truly magnetohydrodynamics tests with $B_{i}\neq0$.

#### RHD Riemann problems

**Figure 4:**
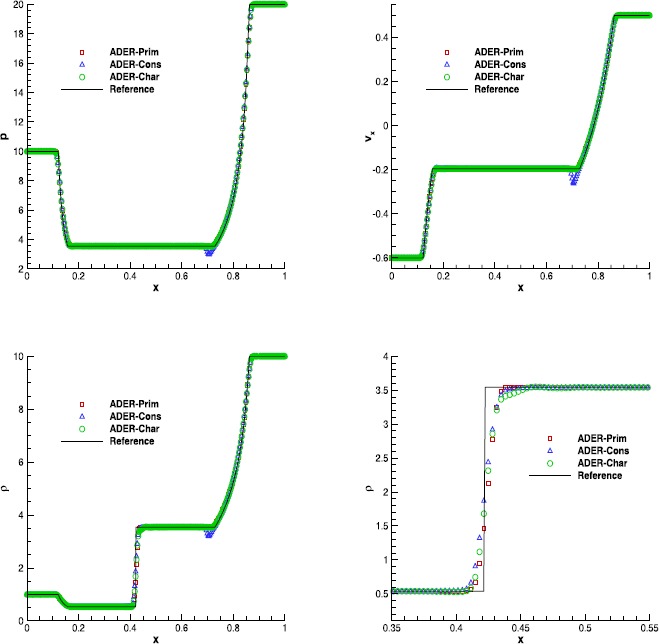
**Solution of RHD-RP1 (see Table** **4** **) with the fourth order ADER-WENO scheme at time** $\pmb{t=0.4}$ **.** The bottom right panel shows a magnification around the contact discontinuity.

**Figure 5:**
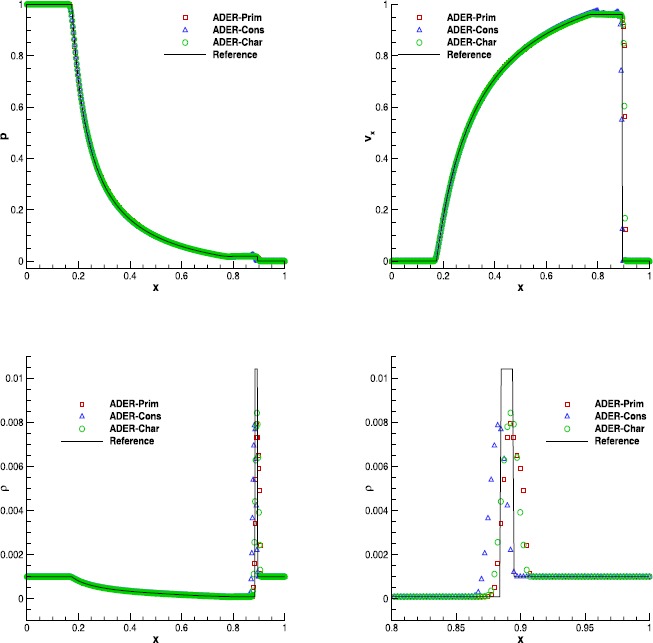
**Solution of RHD-RP2 (see Table** **4** **) with the third order ADER-WENO scheme at time** $\pmb{t=0.4}$ **.** The bottom right panel shows a magnification around the right propagating shock.

Table 4 reports the initial conditions of the two one-dimensional Riemann problems that we have considered, and whose wave-patterns at the final time $t_{f}=0.4$ are shown in Figure 4 and Figure 5, respectively. In order to appreciate the differences among the available ADER implementations, we have again solved each problem with the three alternative schemes: ADER-Prim, ADER-Cons and ADER-Char. The reference solution, computed as in Rezzolla and Zanotti (2001), is shown too.

**Table 4:** **Left and right states of the one-dimensional RHD Riemann problems**

**Problem**		***γ***	***ρ***	$\boldsymbol{v}_{\boldsymbol{x}}$	***p***	$\boldsymbol{t}_{\boldsymbol{f}}$
RHD-RP1	*x*>0	5/3	1	−0.6	10	0.4
	*x* ≤ 0		10	0.5	20	
RHD-RP2	*x*>0	5/3	10^−3^	0.0	1	0.4
	*x* ≤ 0		10^−3^	0.0	10^−5^	

In the first Riemann problem, which was also analyzed by Mignone and Bodo (2005), two rarefaction waves are produced, separated by a contact discontinuity. It has been solved through a fourth order $\mathbb{P}_{0}\mathbb{P}_{3}$ scheme, using the Rusanov Riemann solver over a uniform grid with 300 cells. As it is clear from Figure 4, the ADER-Prim scheme performs significantly better than the ADER-Cons. In particular, the overshoot and undershoot at the tail of the right rarefaction is absent. In general, the results obtained with ADER-Prim are essentially equivalent to those of ADER-Char, namely when the reconstruction in characteristic variables is adopted. This is manifest after looking at the bottom right panel of Figure 4, where a magnification of the rest mass density at the contact discontinuity is shown. Additional interesting comparisons can be made about the second Riemann problem, which can be found in Radice and Rezzolla (2012), and which is displayed in Figure 5. In this case a third order $\mathbb {P}_{0}\mathbb{P}_{2}$ scheme has been used, again with the Rusanov Riemann solver over a uniform grid with 500 cells. The right propagating shock has a strong jump in the rest mass density, as it is visible from the bottom right panel of the figure, and the position of the shock front is better captured by the two schemes ADER-Prim and ADER-Char.

It is particularly interesting to address the issue of CPU time comparison among different implementations of ADER, as already done for the Euler equations. The result of such a comparison, performed for the RHD-RP1 problem, are reported in Table 5, which should be read in synopsis with Table 3. Clearly, ADER-Prim is not only more accurate than ADER-Cons, but it is also more efficient. As anticipated, this is in agreement with our expectations, since in the ADER-Prim implementation a single *cons-to-prim* operation is needed within the cell, rather than at each Gaussian quadrature point and at each space-time degree of freedom. For other tests, see for instance Section 3.2.2, the CPU time reduction implied by ADER-Prim is even more evident, but the numbers shown in Table 5 describe with good fidelity the relative performances of the different ADER in a large number of relativistic tests.

**Table 5:** **CPU time comparison among different ADER implementations for the RHD-RP1 problem**

	**ADER-Prim**	**ADER-Cons**	**ADER-Char**
$\mathbb{P}_{0}\mathbb{P}_{2}$	1.0	1.26	1.40
$\mathbb{P}_{0}\mathbb{P}_{3}$	1.0	1.13	1.24
$\mathbb{P}_{0}\mathbb{P}_{4}$	1.0	1.04	1.06

The numbers have been normalized to the value obtained with ADER-Prim.

#### RHD Kelvin-Helmholtz instability

In the relativistic regime, the Kelvin-Helmholtz (KH) instability is likely to be responsible for a variety of physical effects, which are encountered in the dynamics of extragalactic relativistic jets (Bodo et al. 2004; Perucho et al. 2006; Perucho et al. 2007). As an academic test, we simulate the linear growth phase of the KH instability in two spatial dimensions, taking the initial conditions from Mignone et al. (2009) (see also Beckwith and Stone 2011 and Radice and Rezzolla 2012). In particular, the rest-mass density is chosen as

59 $$ \rho= \textstyle\begin{cases} \rho_{0} + \rho_{1} \tanh{[(y-0.5)/a]}, & y > 0 , \\ \rho_{0} - \rho_{1} \tanh{[(y+0.5)/a]}, & y \leq0 , \end{cases} $$

with $\rho_{0}=0.505$ and $\rho_{1}=0.495$. Assuming that the shear layer has a velocity $v_{s}=0.5$ and a characteristic size $a=0.01$, the velocity along the x-direction is modulated as

60 $$ v_{x} = \textstyle\begin{cases} v_{s} \tanh{[(y-0.5)/a]}, & y > 0 , \\ -v_{s} \tanh{[(y+0.5)/a]}, & y \leq0 . \end{cases} $$

**Figure 6:**
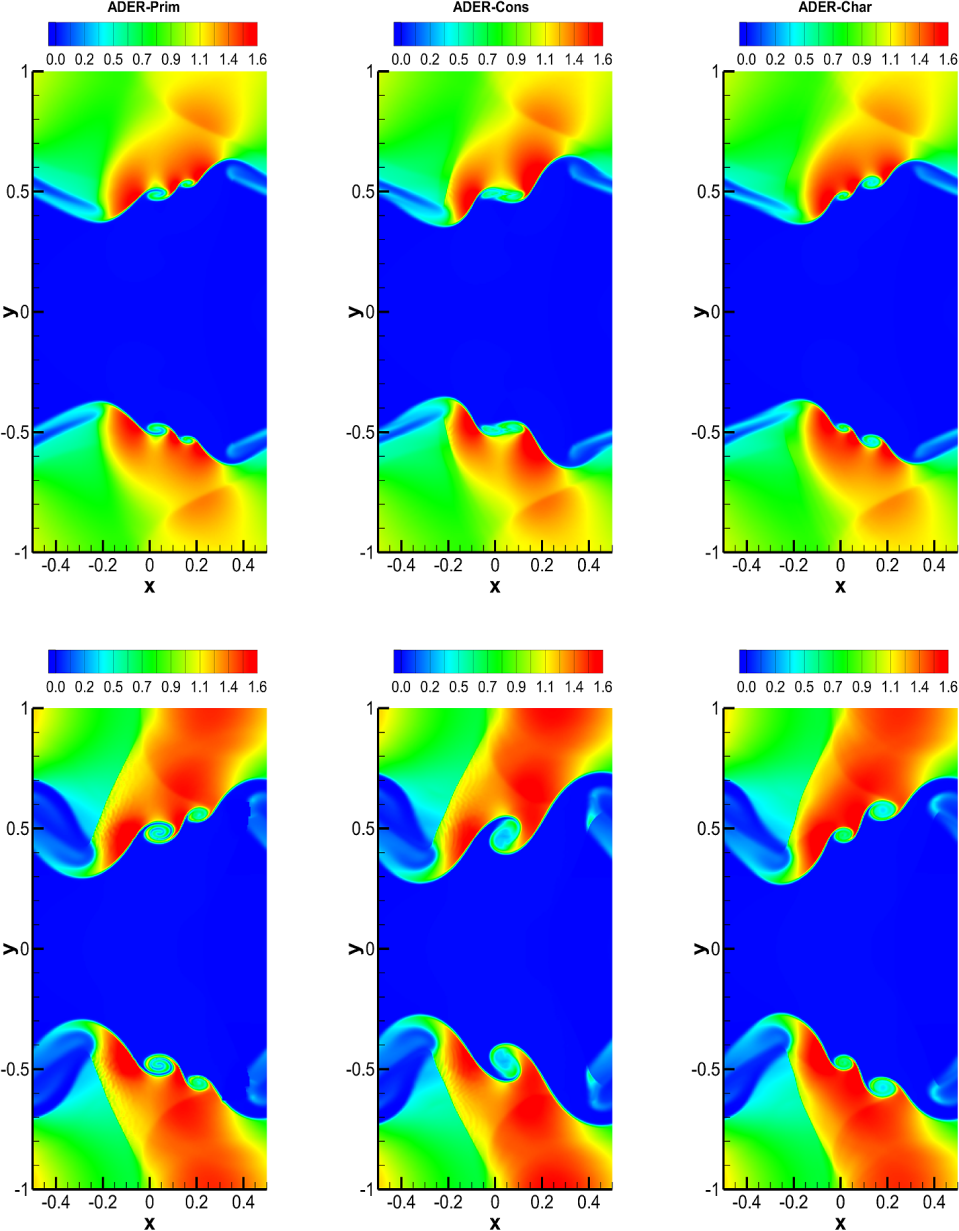
**Two-dimensional Kelvin-Helmholtz instability obtained with the** $\pmb{\mathbb{P}_{0}\mathbb{P}_{3}}$ **scheme and with the Osher flux.** Left panels: solution with ADER-Prim. Central panels: solution with ADER-Cons. Right panels: solution with ADER-Char. Top panels: solution at $t=2.0$. Bottom panels: solution at $t=2.5$.

It is convenient to add a perturbation in the transverse velocity, i.e.

61 $$ v_{y} = \textstyle\begin{cases} \eta_{0} v_{s} \sin (2\pi x) \exp [-(y-0.5)^{2}/\sigma], & y > 0 , \\ -\eta_{0} v_{s} \sin (2\pi x) \exp [-(y+0.5)^{2}/\sigma] , & y \leq0 , \end{cases} $$

where $\eta_{0}=0.1$ is the amplitude of the perturbation, while $\sigma =0.1$ is its length scale. The adiabatic index is $\gamma=4/3$ and the pressure is uniform, $p=1$. The problem has been solved over the computational domain $[-0.5,0.5]\times[-1,1]$, covered by a uniform mesh with $200\times400$ cells, using the $\mathbb{P}_{0}\mathbb{P}_{3}$ scheme and the Osher-type numerical flux. Periodic boundary conditions are fixed both in *x* and in *y* directions. Figure 6 shows the results of the calculations: in the left, in the central and in the right panels we have reported the solution obtained with the ADER-Prim, with the ADER-Cons and with the ADER-Char scheme, respectively, while the top and the bottom panels correspond to two different times during the evolution, namely $t=2.0$ and $t=2.5$. Interestingly, two secondary vortices are visible when the reconstruction is performed in primitive and characteristic variables (see left the right panels), but only one is present in the simulation using the reconstruction in conserved variables. In Zanotti and Dumbser (2015) we have already commented about the elusive character of these details in the solution, which depend both on the resolution and on the Riemann solver adopted. Based on our results, we infer that the ADER-Cons scheme is the most diffusive, while ADER-Prim and ADER-Char seem to produce the same level of accuracy in the solution. However, if we look at the CPU times in the two cases, we find that ADER-Prim is a factor 2.5 faster than ADER-Cons and a factor 3 faster than ADER-Char, and therefore should be preferred in all relevant applications of RHD.

#### RMHD Alfvén wave

In Table 2 of Section 3.1.1 we have reported the comparison of the convergence rates among three different implementations of ADER for the Euler equations. We believe it is important to verify the convergence of the new ADER-Prim scheme also for the RMHD equations, which indeed admits an exact, smooth unsteady solution, namely the propagation of a circularly polarized Alfvén wave (see Komissarov 1997; Del Zanna et al. 2007 for a full account). The wave is assumed to propagate along the *x* direction in a constant density and constant pressure background, say $\rho=p=1$. The magnetic field, on the other hand, is given by

62 $$\begin{aligned}& B_{x} = B_{0}, \end{aligned}$$

63 $$\begin{aligned}& B_{y} = \eta B_{0}\cos\bigl[k(x-v_{A} t)\bigr], \end{aligned}$$

64 $$\begin{aligned}& B_{z} = \eta B_{0}\sin\bigl[k(x-v_{A} t)\bigr] , \end{aligned}$$

where $\eta=1$ is the amplitude of the wave, $B_{0}=1$ is the uniform magnetic field, *k* is the wave number, while $v_{A}$ is speed of propagation of the wave. We have solved this problem over the computational domain $\Omega=[0; 2\pi]\times[0; 2\pi]$, using periodic boundary conditions, the Rusanov Riemann solver and the Adams-Bashforth version for the initial guess of the LSDG predictor. We have compared the numerical solution with the analytic one after one period $T=L/v_{A}=2\pi/v_{A}$. Table 6 contains the results of our analysis, showing the $L_{1}$ and the $L_{2}$ norms of the error of $B^{y}$. As apparent from the table, the nominal order of convergence of the new ADER-Prim scheme is recovered with very good accuracy.

**Table 6:** $\pmb{L_{1}}$ **and** $\pmb{L_{2}}$ **errors analysis for the 2D Alfvén wave problem**

**2D circularly polarized Alfvén wave**						
	$\boldsymbol{N}_{\boldsymbol{x}}$	$\boldsymbol{L}_{\boldsymbol{1}}$ **error**	$\boldsymbol{L}_{\boldsymbol{1}}$ **order**	$\boldsymbol{L}_{\boldsymbol{2}}$ **error**	$\boldsymbol{L}_{\boldsymbol{2}}$ **order**	**Theor.**
$\mathbb{P}_{0}\mathbb{P}_{2}$	50	5.387E-02	-	9.527E-03	-	3
	60	3.123E-02	2.99	5.523E-03	2.99	
	70	1.969E-02	2.99	3.481E-03	2.99	
	80	1.320E-02	2.99	2.334E-03	2.99	
	100	6.764E-03	3.00	1.196E-03	3.00	
$\mathbb{P}_{0}\mathbb{P}_{3}$	50	2.734E-04	-	4.888E-05	-	4
	60	1.153E-04	4.73	2.061E-05	4.74	
	70	5.622E-05	4.66	1.004E-05	4.66	
	80	3.043E-05	4.60	5.422E-06	4.61	
	100	1.108E-05	4.53	1.968E-06	4.54	
$\mathbb{P}_{0}\mathbb{P}_{4}$	30	2.043E-03	-	3.611E-04	-	5
	40	4.873E-04	4.98	8.615E-05	4.98	
	50	1.603E-04	4.98	2.846E-05	4.96	
	60	6.491E-05	4.96	1.168E-05	4.88	
	70	3.173E-05	4.64	6.147E-06	4.16	

The errors have been computed with respect to the magnetic field $B^{y}$.

#### RMHD Riemann problems

**Figure 7:**
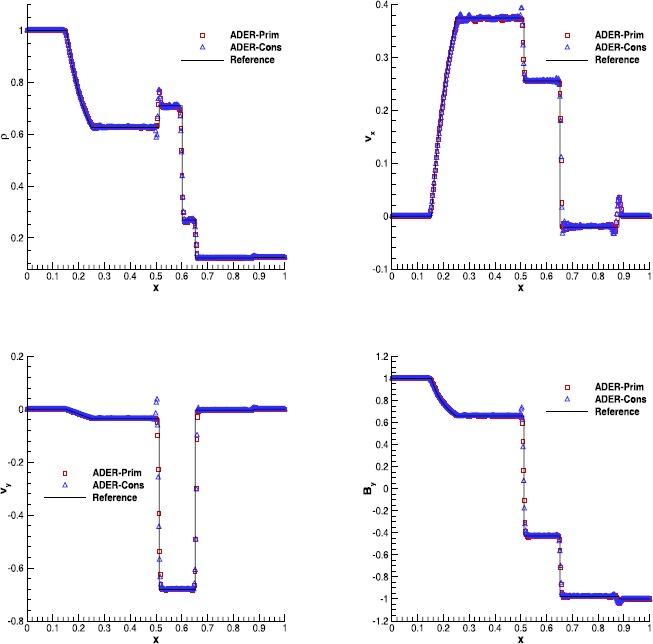
**Solution of RMHD-RP1 (see Table** **7** **) with the fourth order ADER-WENO scheme at time** $\pmb{t=0.4}$ **.** The Rusanov Riemann solver has been used over a 400 cells uniform grid.

**Figure 8:**
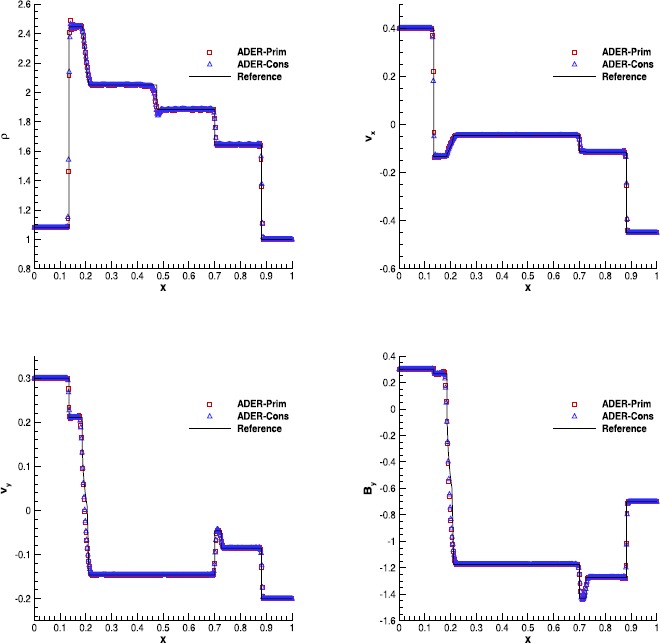
**Solution of RMHD-RP2 (see Table** **7** **) with the fourth order ADER-WENO scheme at time** $\pmb{t=0.55}$ **.** The Rusanov Riemann solver has been used over a 400 cells uniform grid.

Riemann problems are very relevant also in RMHD, admitting a larger number of waves than in hydrodynamics. The exact solution was provided by Giacomazzo and Rezzolla (2006) already ten years ago, making them very popular as a precise tool to validate numerical codes. We have selected Test 1 and Test 5 in Table 1 of Balsara (2001), with initial left and right states that are reported in Table 7. Both the tests have been solved using a fourth order ADER-WENO scheme, the Rusanov Riemann solver and over a uniform grid composed of 400 cells. The damping factor for the divergence-cleaning procedure is set to $\kappa=10$. Figure 7 and Figure 8 allow to compare the exact solution with the results obtained through the ADER-Prim and the ADER-Cons schemes. Especially for RMHD-RP1, the solution obtained with the traditional ADER-Cons scheme is significantly more oscillatory than that produced by ADER-Prim. This is particularly evident in the rest-mass density and in the velocity $v_{x}$. We have here a good indication that the ADER-Prim scheme behaves better than the ADER-Cons scheme when applied to the equations of special relativistic magnetohydrodynamics.

**Table 7:** **Left and right states of the one-dimensional RMHD Riemann problems**

**Problem**		***γ***	***ρ***	$\boldsymbol{(v}_{\boldsymbol{x}}$	$\boldsymbol{v}_{\boldsymbol{y}} $	$\boldsymbol{v}_{\boldsymbol{z}}\boldsymbol{)}$	***p***	$\boldsymbol{(B}_{\boldsymbol{x}} $	$\boldsymbol{B}_{\boldsymbol{y}} $	$\boldsymbol{B}_{\boldsymbol{z}}\boldsymbol{)}$	$\boldsymbol{t}_{\boldsymbol{f}}$
RMHD-RP1	*x*>0	2.0	0.125	0.0	0.0	0.0	0.1	0.5	−1.0	0.0	0.4
	*x* ≤ 0		1.0	0.0	0.0	0.0	1.0	0.5	1.0	0.0	
RMHD-RP2	*x*>0	5/3	1.0	−0.45	−0.2	0.2	1.0	2.0	−0.7	0.5	0.55
	*x* ≤ 0		1.08	0.4	0.3	0.2	0.95	2.0	0.3	0.3	

#### RMHD rotor problem

The relativistic version of the magnetic rotor problem, originally proposed by Balsara and Spicer (1999), has by now become a standard numerical test in RMHD. It describes the evolution of a high density plasma which, at time $t=0$, rotates rapidly with angular velocity *ω* and is surrounded by a low density plasma at rest:

65 $$\begin{aligned}& \rho=\textstyle\begin{cases} 10 & \text{for } 0\le r\le0.1; \\ 1 & \text{otherwise}; \end{cases}\displaystyle \\& \omega=\textstyle\begin{cases} 9.3 & \text{for } 0\le r\le0.1; \\ 0 & \text{otherwise}; \end{cases}\displaystyle \quad\quad\mathbf{B} = \begin{pmatrix} 1.0 \\ 0 \\ 0 \end{pmatrix}, \\& p = 1 ,\quad\quad\gamma=4/3. \end{aligned}$$

**Figure 9:**
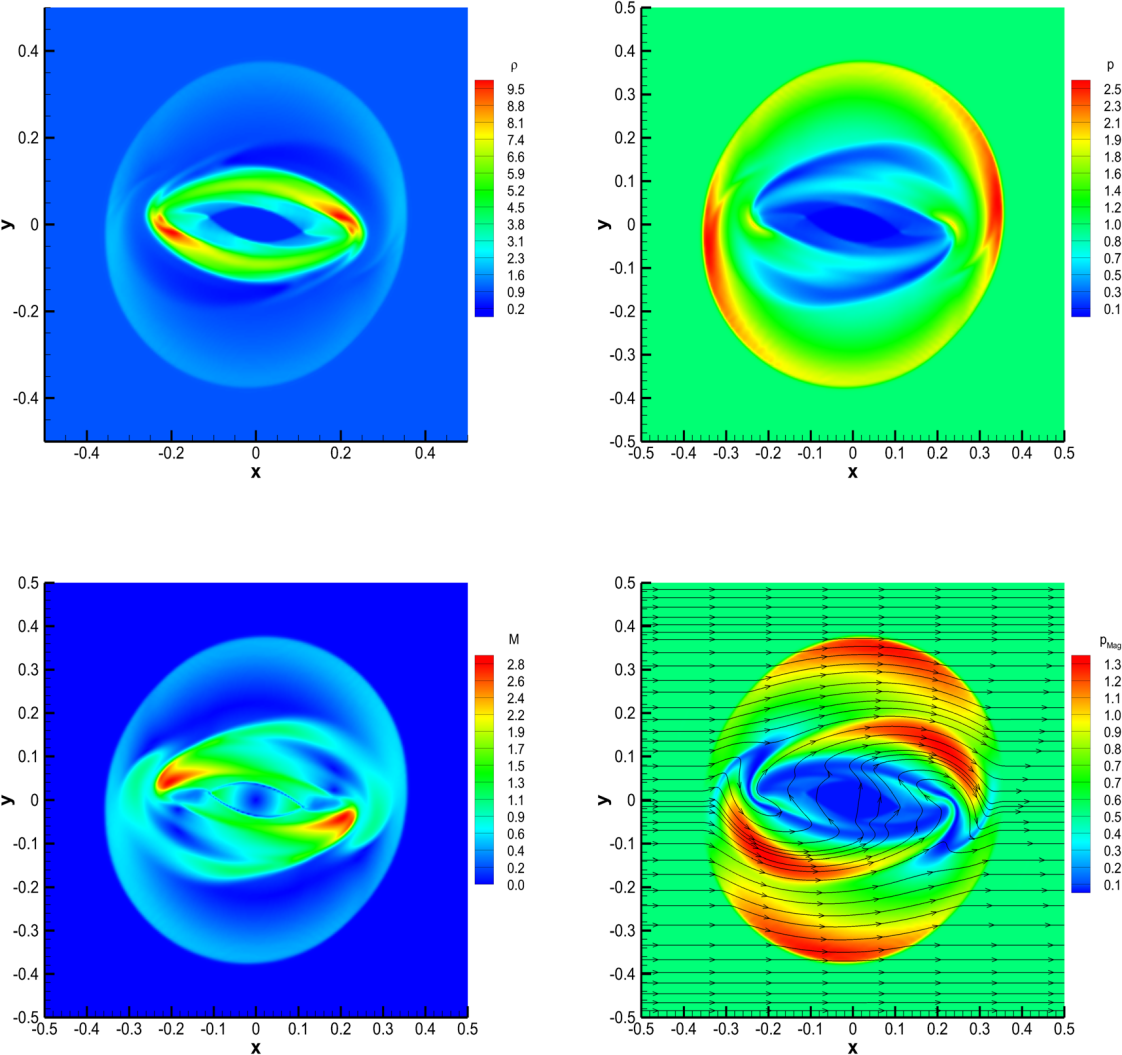
**Solution of the RMHD rotor problem at time** $\pmb{t=0.4}$ **, obtained with the** $\pmb{\mathbb{P}_{0}\mathbb{P}_{3}}$ **scheme on a uniform grid with** $\pmb{300\times300}$ **cells.** Top panels: rest-mass density (left) and thermal pressure (right). Bottom panels: Mach number (left) and magnetic pressure (right).

Due to rotation, a sequence of torsional Alfvén waves are launched outside the cylinder, with the net effect of reducing the angular velocity of the rotor. We have solved this problem over a computational domain $\Omega= [-0.6,0.6]\times [-0.6,0.6]$, discretized by $300\times300$ numerical cells and using a fourth order finite volume scheme with the Rusanov Riemann solver. No taper has been applied to the initial conditions, thus producing true discontinuities right at the beginning. Figure 9 shows the rest-mass density, the thermal pressure, the relativistic Mach number and the magnetic pressure at time $t=0.4$. We obtain results which are in good qualitative agreement with those available in the literature (see, for instance, Del Zanna et al. 2003; Dumbser and Zanotti 2009; Loubère et al. 2014 and Kim and Balsara 2014). We emphasize that for this test the reconstruction of the primitive variables $v^{i}$ turns out to be more robust than that achieved through the reconstruction of the products $Wv^{i}$.

### The Baer-Nunziato equations

As a genuinely non-conservative system of hyperbolic equations we consider the Baer-Nunziato model for compressible two-phase flow (see also Baer and Nunziato 1986; Saurel and Abgrall 1999; Andrianov and Warnecke 2004; Schwendeman et al. 2006; Deledicque and Papalexandris 2007; Murrone and Guillard 2005). In the rest of the paper we define the first phase as the solid phase and the second phase as the gas phase. As a result, we will use the subscripts 1 and *s* as well as 2 and *g* as synonyms. Sticking to Baer and Nunziato (1986), we prescribe the interface velocity $\mathbf{v}_{I}$ and the pressure $p_{I}$ as $\mathbf{v}_{I} = \mathbf{v}_{1}$ and $p_{I} = p_{2}$, respectively, although other choices are also possible (Saurel and Abgrall 1999). With these definitions, the system of Baer-Nunziato equations can be cast in the form prescribed by (1) after defining the state vector **Q** as

66 $$\begin{aligned} \mathbf{Q} =& \bigl( \phi _{1}\rho _{1}, \phi _{1}\rho _{1}v_{1}^{i}, \phi _{1}\rho _{1}E_{1}, \\ &\phi _{2}\rho _{2}, \phi _{2}\rho _{2}v_{2}^{i}, \phi _{2}\rho _{2}E_{2}, \phi_{1} \bigr) , \end{aligned}$$

where $\phi_{k}$ is the volume fraction of phase *k*, with the condition that $\phi_{1}+\phi_{2}=1$. On the other hand, the fluxes $\mathbf{f}^{i}$, the sources **S** and the non-conservative matrices $\mathbf{B}_{i}$ are expressed by

67 $$\begin{aligned}& \begin{aligned} &\mathbf{f}^{i}=\begin{bmatrix} \phi _{1}\rho _{1}v_{1}^{i} \\ \phi_{1}( \rho_{1} v_{1}^{i} v_{1}^{j} + p_{1}\delta^{ij} ) \\ \phi_{1} v_{1}^{i}(\rho_{1} E_{1}+p_{1}) \\ \phi_{2}\rho_{2} v_{2}^{i} \\ \phi_{2}( \rho_{2} v_{2}^{i} v_{2}^{j} + p_{2}\delta^{ij} )\\ \phi_{2} v_{2}^{i}(\rho_{2} E_{2} + p_{2}) \\ 0 \end{bmatrix}, \\ &\mathbf{S}=\begin{bmatrix} 0 \\ -\nu(v_{1}^{i} - v_{2}^{i}) \\ -\nu\mathbf{v}_{1} \cdot(\mathbf{v}_{1} - \mathbf{v}_{2}) \\ 0 \\ -\nu (v_{2}^{i} - v_{1}^{i})\\ -\nu\mathbf{v}_{1} \cdot(\mathbf{v}_{2} - \mathbf{v}_{1}) \\ \mu (p_{1}-p_{2}) \end{bmatrix} , \end{aligned} \end{aligned}$$

68 $$\begin{aligned}& \mathbf{B} _{i} = \left (\textstyle\begin{array}{c@{\quad}c@{\quad}c@{\quad}c@{\quad}c@{\quad}c@{\quad}c@{\quad}c@{\quad}c@{\quad}c@{\quad}c} 0&0&0&0&0&0&0&0&0&0&0\\ 0&0&0&0&0&0&0&0&0&0& - p_{I} \mathbf{e}_{i} \\ 0&0&0&0&0&0&0&0&0&0& - p_{I} v_{I}^{i}\\ 0&0&0&0&0&0&0&0&0&0&0\\ 0&0&0&0&0&0&0&0&0&0&p_{I} \mathbf{e}_{i} \\ 0&0&0&0&0&0&0&0&0&0&p_{I} v_{I}^{i}\\ 0&0&0&0&0&0&0&0&0&0&v_{I}^{i} \end{array}\displaystyle \right ), \end{aligned}$$

where $\mathbf{e}_{i}$ is the unit vector pointing in direction *i* (${i \in \{x,y,z \}}$) and *ν* and *μ* are two parameters related to the friction between the phases and to the pressure relaxation.^5^

**Figure 10:**
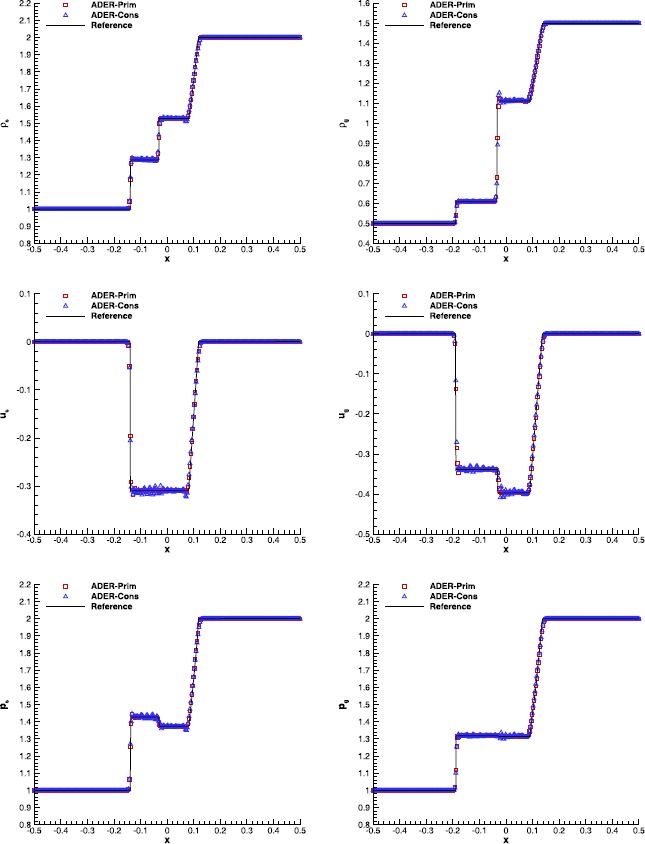
**Results for the Baer-Nunziato Riemann problem BNRP1.** The Osher Riemann solver has been used over a 300 cells uniform grid.

**Figure 11:**
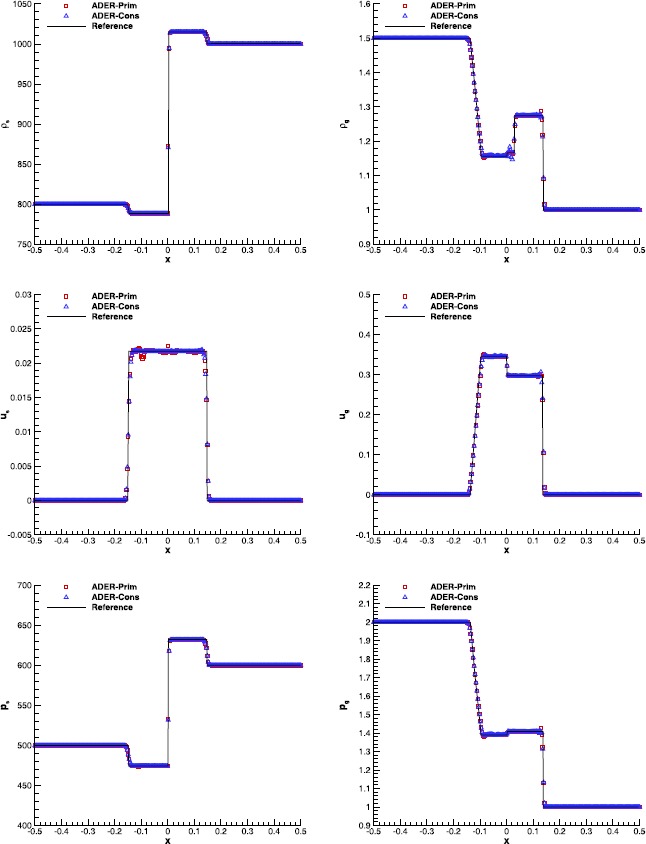
**Results for the Baer-Nunziato Riemann problem BNRP2.** The Osher Riemann solver has been used over a 300 cells uniform grid.

**Figure 12:**
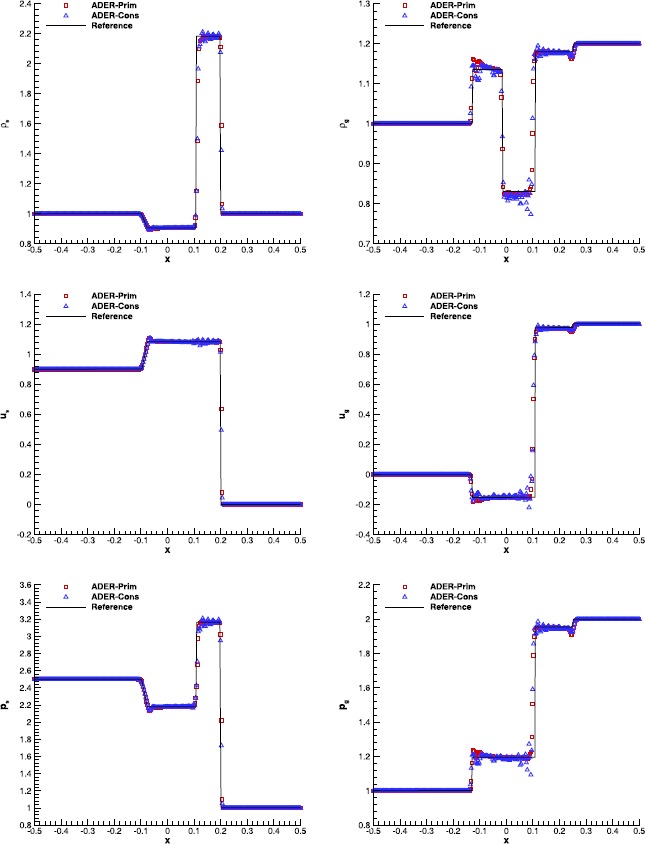
**Results for the Baer-Nunziato Riemann problem BNRP3.** The Osher Riemann solver has been used over a 300 cells uniform grid.

**Figure 13:**
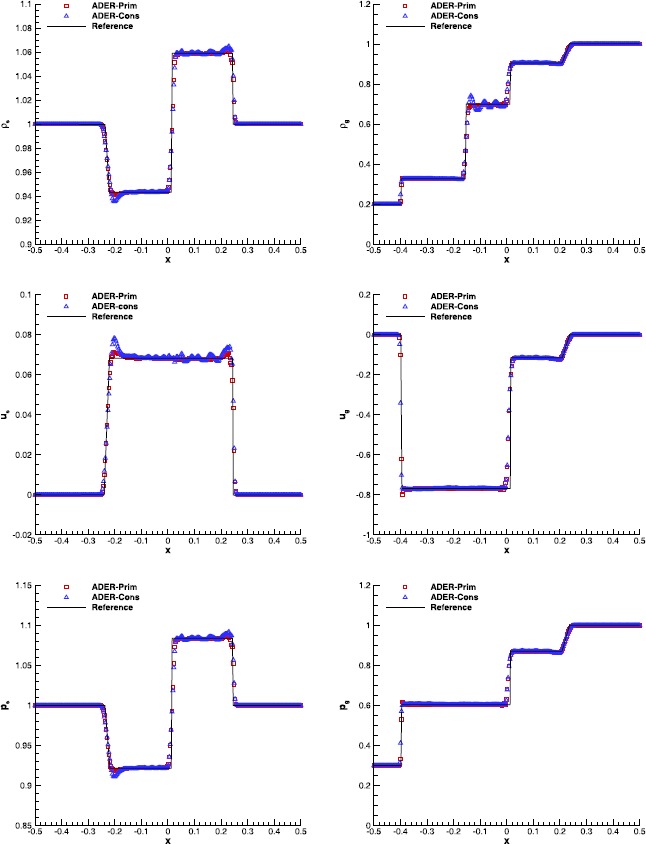
**Results for the Baer-Nunziato Riemann problem BNRP5.** The Rusanov Riemann solver has been used over a 300 cells uniform grid.

**Figure 14:**
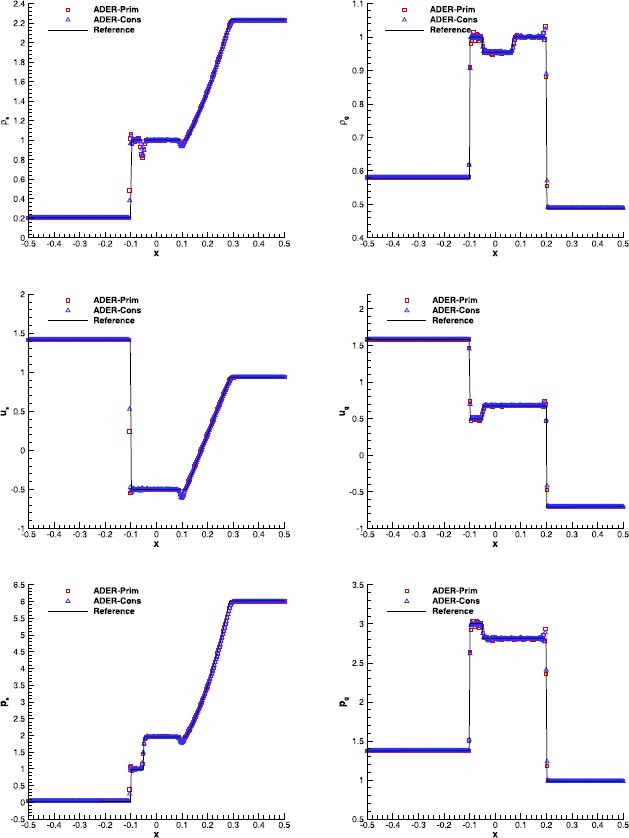
**Results for the Baer-Nunziato Riemann problem BNRP6.** The Osher Riemann solver has been used over a 300 cells uniform grid.

The equation of state is the so-called stiffened gas equation of state,

69 $$ \epsilon_{k} = \frac{p_{k} + \gamma_{k} \pi_{k}}{\rho_{k} (\gamma_{k} -1 )} , $$

which is a simple modification of the ideal gas EOS and where $\pi_{k}$ expresses a reference pressure. For brevity, we have solved this system of equations only for a set of one-dimensional Riemann problems, with initial conditions reported in Table 8. The name of the models, BNRP1, BNRP2, etc., respects the numeration adopted in Dumbser et al. (2010). A reference solution is available for these tests, and it can be found in Andrianov and Warnecke (2004), Schwendeman et al. (2006), Deledicque and Papalexandris (2007). Each Riemann problem has been solved using a fourth order WENO scheme with 300 cells uniformly distributed over the range $[-0.5;0.5]$. In Figures 10-14 we have reported the comparison among the solutions obtained with the ADER-Prim, with the ADER-Cons and with the exact solver. In all the tests, with the exception of BNRP2, the ADER-Prim scheme behaves significantly better than the ADER-Cons scheme. On several occasions, such as for $v_{s}$ and $v_{g}$ in BNRP1, or for most of the quantities in BNRP5, the solution provided through ADER-Cons manifest evident oscillations, which are instead strongly reduced, or even absent, when the ADER-Prim scheme is used. The CPU time overhead implied by ADER-Prim is comparatively limited, and never larger than ∼20 %.

**Table 8:** **Initial states left (L) and right (R) for the Riemann problems for the Baer-Nunziato equations**

	$\boldsymbol{\rho}_{\boldsymbol{s}}$	$\boldsymbol{u}_{\boldsymbol{s}}$	$\boldsymbol{p}_{\boldsymbol{s}}$	$\boldsymbol{\rho}_{\boldsymbol{g}}$	$\boldsymbol{u}_{\boldsymbol{g}}$	$\boldsymbol{p}_{\boldsymbol{g}}$	$\boldsymbol{\phi}_{\boldsymbol{s}}$	$\boldsymbol{t}_{\boldsymbol{e}}$
BNRP1 (Deledicque and Papalexandris 2007): $\gamma_{s} = 1.4$, $\pi_{s} = 0$, $\gamma _{g} = 1.4$, $\pi_{g} = 0$								
L	1.0	0.0	1.0	0.5	0.0	1.0	0.4	0.10
R	2.0	0.0	2.0	1.5	0.0	2.0	0.8	
BNRP2 (Deledicque and Papalexandris 2007): $\gamma_{s} = 3.0$, $\pi_{s} = 100$, $\gamma_{g} = 1.4$, $\pi_{g} = 0$								
L	800.0	0.0	500.0	1.5	0.0	2.0	0.4	0.10
R	1,000.0	0.0	600.0	1.0	0.0	1.0	0.3	
BNRP3 (Deledicque and Papalexandris 2007): $\gamma_{s} = 1.4$, $\pi_{s} = 0$, $\gamma _{g} = 1.4$, $\pi_{g} = 0$								
L	1.0	0.9	2.5	1.0	0.0	1.0	0.9	0.10
R	1.0	0.0	1.0	1.2	1.0	2.0	0.2	
BNRP5 (Schwendeman et al. 2006): $\gamma_{s} = 1.4$, $\pi_{s} = 0$, $\gamma _{g} = 1.4$, $\pi_{g} = 0$								
L	1.0	0.0	1.0	0.2	0.0	0.3	0.8	0.20
R	1.0	0.0	1.0	1.0	0.0	1.0	0.3	
BNRP6 (Andrianov and Warnecke 2004): $\gamma_{s} = 1.4$, $\pi_{s} = 0$, $\gamma _{g} = 1.4$, $\pi_{g} = 0$								
L	0.2068	1.4166	0.0416	0.5806	1.5833	1.375	0.1	0.10
R	2.2263	0.9366	6.0	0.4890	−0.70138	0.986	0.2	

Values for $\gamma_{i}$, $\pi_{i}$ and the final time $t_{e}$ are also reported.

## Extension to discontinuous Galerkin and adaptive mesh refinement

Although we have so far concentrated on the implementation of the new ADER-Prim scheme in the context of finite volume methods, the same idea can be extended to discontinuous Galerkin (DG) schemes as well. Incidentally, we note that the interest of computational astrophysics towards DG methods is increasing (Radice and Rezzolla 2011; Teukolsky 2015), and, especially in the relativistic context, they are expected to play a crucial role in the years to come. In a sequence of papers, we have recently developed a class of robust DG schemes which are able to cope even with discontinuous solutions, by incorporating an a posteriori subcell limiter (Dumbser et al. 2014; Zanotti et al. 2015b; Zanotti et al. 2015a). The whole logic can be briefly summarized as follows. First we assume a *discrete representation* of the solution, in conserved variables, at any given time $t^{n}$ as

70 $$ \mathbf{u}_{h}\bigl(\mathbf{x},t^{n}\bigr) = \sum_{l=0}^{N}\Phi_{l}( \boldsymbol{\xi}) \hat {\mathbf{u}}^{n}_{l}= \Phi_{l}(\boldsymbol{\xi}) \hat{\mathbf{u}}^{n}_{l},\quad \mathbf{x}\in T_{i} , $$

in which the polynomials

71 $$ \Phi_{l}(\boldsymbol{\xi}) = \psi_{p}(\xi) \psi_{q}(\eta) \psi _{r}(\zeta) $$

are built using the spatial Lagrange interpolation polynomials already adopted for the WENO reconstruction. The time evolution of the *degrees of freedom* $\hat{\mathbf {u}}^{n}_{l}$ is then obtained after considering the weak form of the governing PDE, which leads to

72 $$\begin{aligned}& \biggl( \int _{T_{i}} \Phi_{k} \Phi_{l}\, d\mathbf{x} \biggr) \bigl( \hat {\mathbf{u}}_{l}^{n+1} - \hat{ \mathbf{u}}_{l}^{n} \bigr) \\& \quad\quad{}+ \int _{t^{n}}^{t^{n+1}} \int _{\partial T_{i}} \Phi_{k} \biggl( \tilde{\mathbf{ f}} \bigl(\mathbf{v}_{h}^{-}, \mathbf{v}_{h}^{+} \bigr) + \frac{1}{2} \mathcal {D} \bigl(\mathbf{v}_{h}^{-}, \mathbf{v}_{h}^{+} \bigr) \biggr) \cdot\mathbf{n} \,dS \,dt \\& \quad\quad{} - \int _{t^{n}}^{t^{n+1}} \int _{T_{i}} \nabla\Phi_{k} \cdot \mathbf{F} ( \mathbf{v}_{h} )\, d\mathbf{x} \,dt \\& \quad\quad{}+ \int _{t^{n}}^{t^{n+1}} \int _{T_{i}} \Phi_{k}\mathbf{B}(\mathbf{v}_{h}) \cdot\mathbf{M}\nabla\mathbf{v}_{h}\, d\mathbf{x} \,dt \\& \quad= \int _{t^{n}}^{t^{n+1}} \int _{T_{i}} \Phi_{k} \mathbf{S} ( \mathbf{v}_{h} ) \, d\mathbf{x} \,dt , \end{aligned}$$

where, just like in Eq. (22), $\tilde{\mathbf{ f}}$ denotes a numerical flux function and $\mathcal{D} (\mathbf {v}_{h}^{-}, \mathbf{v}_{h}^{+} )$ a path-conservative jump term. Obviously, no spatial WENO reconstruction is needed within the DG framework, and the local spacetime DG predictor $\mathbf{v}_{h}(\mathbf{x},t)$ entering Eq. (72) will be computed according to the same strategy outlined in Section 2.4.1. T although acting directly over the degrees of freedom $\hat{\mathbf {p}}^{n}_{l}$ in primitive variables, which are computed from the degrees of freedom $\hat{\mathbf{u}}^{n}_{l}$ in conserved variables simply by

73 $$ \hat{\mathbf{p}}^{n}_{l} = \mathbf{V} \bigl( \hat{ \mathbf{Q}}^{n}_{l} \bigr),\quad \forall l. $$

The conversion can be done in such a simple way because we use a *nodal* basis $\Phi_{l}(\mathbf{x})$. In other words, the degrees of freedom $\hat{\mathbf{u}}^{n}_{l}$ in conserved variables are first converted into degrees of freedom $\hat {\mathbf{p} }^{n}_{l}$ in primitive variables, which are then used as initial conditions for the LSDG predictor, i.e.

74 $$\begin{aligned}& {\mathbf{u}}_{h}\bigl(\mathbf{x},t^{n}\bigr) \xrightarrow{\mathit{Cons}2\mathit{Prim}} {\mathbf{p} }_{h}\bigl(\mathbf {x},t^{n}\bigr)\xrightarrow{\mathit{LSDG}} {\mathbf{v}}_{h}( \mathbf{x},t) , \\& \quad t\in \bigl[t^{n};t^{n+1}\bigr] . \end{aligned}$$

In those cells in which the main scheme of Eq. (72) fails, either because unphysical values of any quantity are encountered, or because strong oscillations appear in the solution which violate the discrete maximum principle, the computation within the troubled cell goes back to the time level $t^{n}$ and it proceeds to a complete re-calculation. In practice, a suitable subgrid is generated just within the troubled cell, and a traditional finite volume scheme is used on the subgrid using an alternative data representation in terms of cell averages defined for each cell of the subgrid. This approach and the underlying *a posteriori* MOOD framework have been presented in full details in Clain et al. (2011), Diot et al. (2012), Dumbser et al. (2014), to which we address the interested reader for a deeper understanding.

The resulting ADER-DG scheme in primitive variables can be combined with spacetime adaptive mesh refinement (AMR), in such a way to resolve the smallest details of the solution in highly complex flows. We refer to Zanotti et al. (2015b), Zanotti et al. (2015a) for a full account of our AMR solver in the context of ADER-DG schemes. Here we want to show three representative test cases of the ability of the new ADER-Prim-DG scheme with adaptive mesh refinement, by considering the cylindrical expansion of a blast wave in a plasma with an initially uniform magnetic field (see also Komissarov 1999; Leismann et al. 2005; Del Zanna et al. 2007; Dumbser and Zanotti 2009), as well as the shock problems of Leblanc, Sedov (1959) and Noh (1987).

### RMHD blast wave problem

At time $t=0$, the rest-mass density and the pressure are $\rho=0.01$ and $p=1$, respectively, within a cylinder of radius $R=1.0$, while outside the cylinder $\rho=10^{-4}$ and $p=5\times10^{-4}$. Moreover, there is a constant magnetic field $B_{0}$ along the *x*-direction and the plasma is at rest, while a smooth ramp function between $r=0.8$ and $r=1$ modulates the initial jump between inner and outer values, similarly to Komissarov (1999) and Del Zanna et al. (2007).

**Figure 15:**
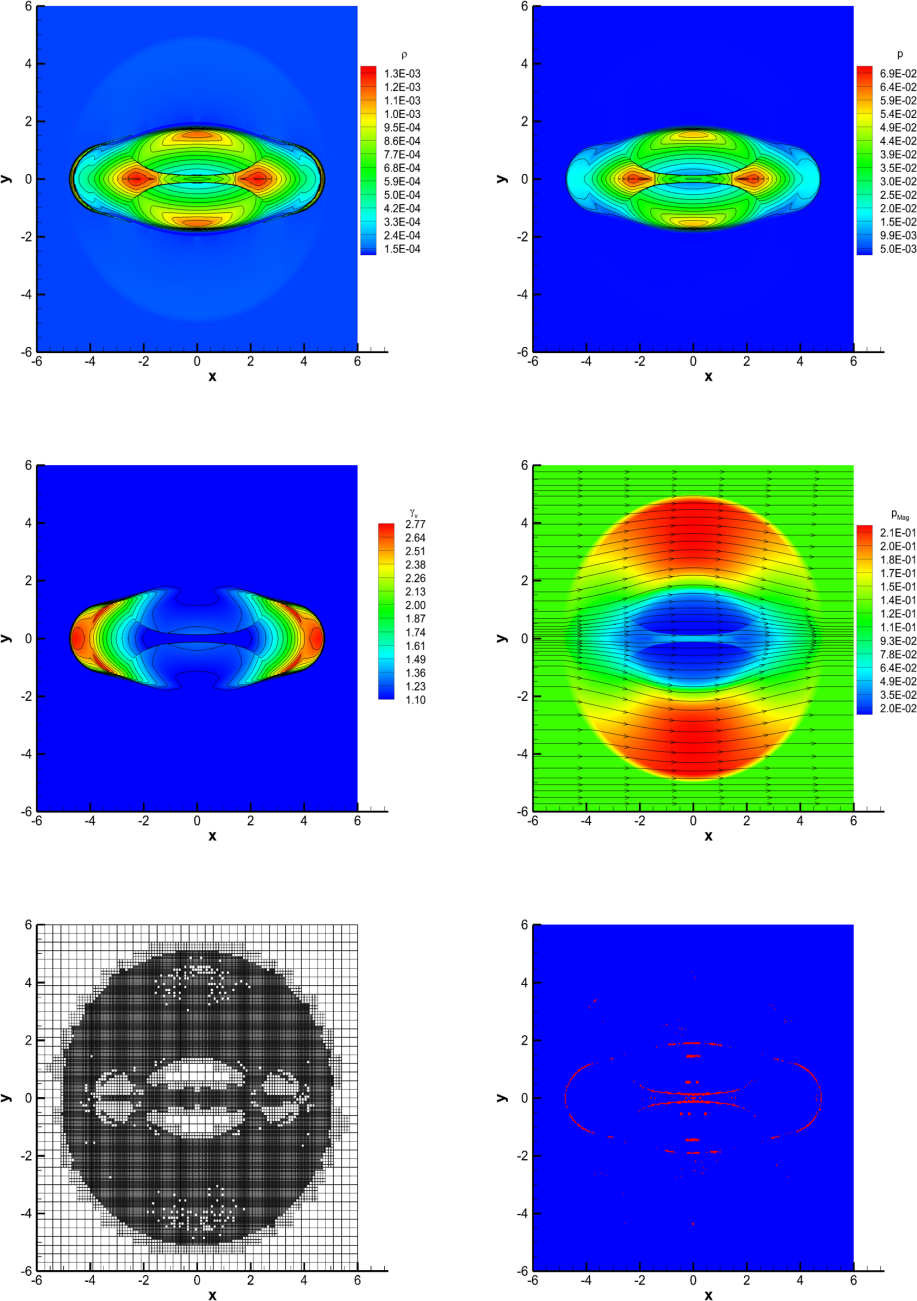
**Solution of the RMHD blast wave at time** $\pmb{t=4.0}$ **, obtained with the ADER-DG** $\pmb{\mathbb{P}_{3}\mathbb{P}_{3}}$ **scheme supplemented with the** ***a posteriori*** **second order TVD subcell finite volume limiter.** Top panels: rest-mass density (left) and thermal pressure (right). Central panels: Lorentz factor (left) and magnetic pressure (right), with magnetic field lines reported. Bottom panels: AMR grid (left) and limiter map (right) with troubled cells marked in red and regular unlimited cells marked in blue.

The computational domain is $\Omega= [-6,6]\times[-6,6]$, and the problem has been solved over an initial coarse mesh with $40\times40$ elements. During the evolution the mesh is adaptively refined using a refinement factor along each direction $\mathfrak{r}=3$ and two levels of refinement. A simple Rusanov Riemann solver has been adopted, in combination with the $\mathbb{P}_{3}\mathbb{P}_{3}$ version of the ADER-DG scheme. On the subgrid we are free to choose any finite volume scheme that we wish, and for this specific test we have found convenient to adopt a second-order TVD scheme. The results for $B_{0}=0.5$ are shown in Figure 15, which reports the rest-mass density, the thermal pressure, the Lorentz factor and the magnetic pressure at time $t=4.0$. At this time, the solution is composed by an external circular fast shock wave, which is hardly visible in the rest mass density, and a reverse shock wave, which is compressed along the *y*-direction. The magnetic field is mostly confined between these two waves, as it can be appreciated from the contour plot of the magnetic pressure. The two bottom panels of the figure show the AMR grid (bottom left) and the map of the limiter (bottom right). In the latter we have used the red color to highlight those cells which required the activation of the limiter over the subgrid, while the blue color is for the regular cells. In practice, the limiter is only needed at the inner shock front, while the external shock front is so weak that the limiter is only occasionally activated. These results confirm the ability of the new ADER-Prim scheme to work also in combination with discontinuous Galerkin methods, and with complex systems of equations like RMHD.

### Leblanc, Sedov and Noh problem

**Figure 16:**
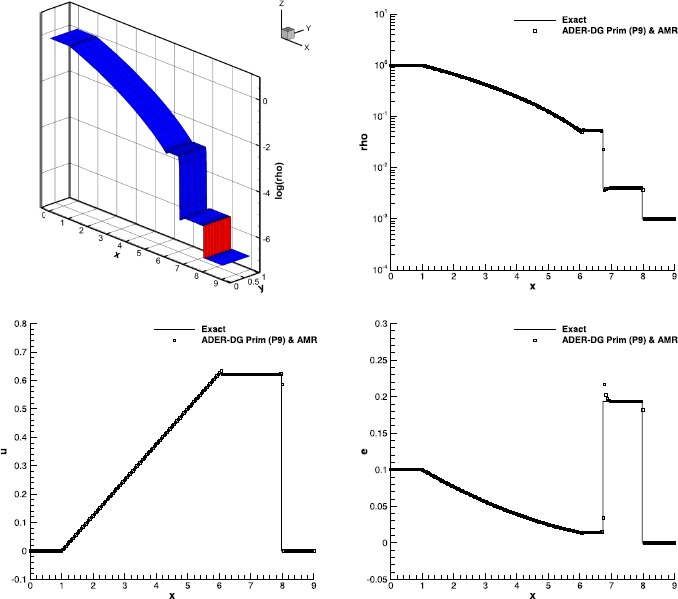
**Solution of the Leblanc shock tube problem at time** $\pmb{t=6.0}$ **, obtained with the ADER-DG** $\pmb{\mathbb{P}_{9}\mathbb{P}_{9}}$ **scheme supplemented with the** ***a posteriori*** **second order TVD subcell finite volume limiter.** Top left: Troubled cells highlighted in red and unlimited cells in blue. Top right to bottom right: Comparison with the exact solution using a 1D cut through the 2D solution on 200 equidistant sample points for density, velocity and internal energy.

**Figure 17:**
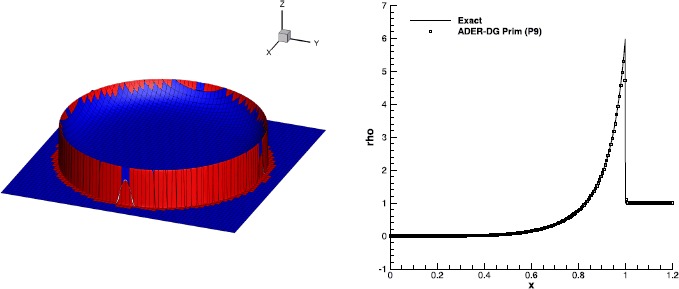
**Solution of the Sedov problem at time** $\pmb{t=1.0}$ **, obtained with the ADER-DG** $\pmb{\mathbb{P}_{9}\mathbb{P}_{9}}$ **scheme supplemented with the** ***a posteriori*** **second order TVD subcell finite volume limiter.** Left: Troubled cells highlighted in red and unlimited cells in blue. Right: Comparison with the exact solution along the *x*-axis.

**Figure 18:**
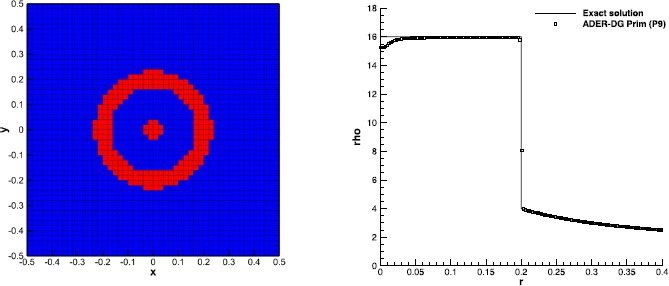
**Solution of the Noh problem at time** $\pmb{t=0.6}$ **, obtained with the ADER-DG** $\pmb{\mathbb{P}_{9}\mathbb{P}_{9}}$ **scheme supplemented with the** ***a posteriori*** **second order TVD subcell finite volume limiter.** Left: Troubled cells highlighted in red and unlimited cells in blue. Right: Comparison with the exact solution along the *x*-axis.

Here we solve again the classical Euler equations of compressible gas dynamics on a rectangular domain for the Leblanc problem and on a circular domain in the case of the shock problems of Sedov and Noh. The initial conditions are detailed in Dumbser et al. (2013), Boscheri et al. (2014b), Boscheri and Dumbser (2014). For the low pressure region that is present in the above test problems, we use $p=10^{-14}$ for the Leblanc and the Noh problem. The computational results obtained with very high order ADER-DG $\mathbb{P}_{9}\mathbb{P}_{9}$ schemes are depicted in Figures 16, 17 and 18, showing an excellent agreement with the exact solution in all cases, apart from the overshoot in the case of the Leblanc shock tube. We stress that all test problems are extremely severe and therefore clearly demonstrate the robustness of the new approach.

## Conclusions

The new version of ADER schemes introduced in Dumbser et al. (2008b) relies on a local space-time discontinuous Galerkin predictor, which is then used for the computation of high order accurate fluxes and sources. This approach has the advantage over classical Cauchy-Kovalewski based ADER schemes (Toro et al. 2001; Titarev and Toro 2002; Toro and Titarev 2002; Titarev and Toro 2005; Toro and Titarev 2006; Dumbser and Munz 2006; Taube et al. 2007) that it is in principle applicable to general nonlinear systems of conservation laws. However, for hyperbolic systems in which the conversion from conservative to primitive variables is not analytic but only available numerically, a large number of such expensive conversions must be performed, namely one for each space-time quadrature point for the integration of the numerical fluxes over the element interfaces and one for each space-time degree of freedom in the local space-time DG predictor.

Motivated by this limitation, we have designed a new version of ADER schemes, valid primarily for finite volume schemes but extendible also to the discontinuous Galerkin finite element framework, in which both the spatial WENO reconstruction and the subsequent local space-time DG predictor act on the primitive variables. In the finite volume context this can be done by performing a double WENO reconstruction for each cell. In the first WENO step, piece-wise polynomials of the conserved variables are computed from the cell averages in the usual way. Then, these reconstruction polynomials are simply *evaluated* in the cell centers, in order to obtain *point values* of the conserved variables. After that, a single conversion from the conserved to the primitive variable is needed in each cell. Finally, a second WENO reconstruction acts on these point values and provides piece-wise polynomials of the primitive variables. The local space-time discontinuous Galerkin predictor must then be reformulated in a non-conservative fashion, supplying the time evolution of the reconstructed polynomials for the primitive variables.

For all systems of equations that we have explored, classical Euler, relativistic hydrodynamics (RHD) and magnetohydrodynamics (RMHD) and the Baer-Nunziato equations, we have noticed a significant reduction of spurious oscillations provided by the new reconstruction in primitive variables with respect to traditional reconstruction in conserved variables. This effect is particularly evident for the Baer-Nunziato equations. In the relativistic regime, there is also an improvement in the ability of capturing the position of shock waves (see Figure 5). To a large extent, the new primitive formulation provides results that are comparable to reconstruction in characteristic variables.

Moreover, for systems of equations in which the conversion from the conserved to the primitive variables cannot be obtained in closed form, such as for the RHD and RMHD equations, there is an advantage in terms of computational efficiency, with reductions of the CPU time around ∼20 %, or more. We have also introduced an additional improvement, namely the implementation of a new initial guess for the LSDG predictor, which is based on an extrapolation in time, similar to Adams-Bashforth-type ODE integrators. This new initial guess is typically faster than those traditionally available, but it is also less robust in the presence of strong shocks.

We predict that the new version of ADER based on primitive variables will become the standard ADER scheme in the relativistic framework. This may become particularly advantageous for high energy astrophysics, in which both high accuracy and high computational efficiency are required.

## Footnotes

1 Since we adopt Cartesian coordinates, $\mathbf{ f}^{x}(\mathbf{Q})$, $\mathbf{f}^{y}(\mathbf{Q})$, $\mathbf{f}^{z}(\mathbf{Q})$ express the fluxes along the *x*, *y* and *z* directions, respectively.

2 See Müller et al. (2013) for more sophisticated paths.

3 See Appendix A of Zanotti and Dumbser (2015) for a graphical representation.

4 We note that, since the spacetime is flat and we are using Cartesian coordinates, the covariant and the contravariant components of spatial vectors can be used interchangeably, namely $A_{i}=A^{i}$, for the generic vector *A⃗*.

5 In the tests below *ν* and *μ* are both set to zero.
